# Supplementary material for: Evaluating the performance of artificial intelligence software for lung nodule detection on chest radiographs in a retrospective real-world UK population
Source: BMJ Open. 2023 Nov 8;13(11):e077348. doi: 10.1136/bmjopen-2023-077348 (PMC10632826; doi:10.1136/bmjopen-2023-077348)
Supplement: Supplementary data [file bmjopen-2023-077348supp001.pdf]

***A**. accuracy of nodule detection by software*		**Clinical report** REFERENCE STANDARD	
		**Positive**	**Negative**
**ALND software** TEST	**Positive**	55 TRUE POSITIVE	946 FALSE POSITIVE
	**Negative**	45 FALSE NEGATIVE	4676 TRUE NEGATIVE

***B**. accuracy of cancer detection by software*		**MDT-reported cancer** REFERENCE STANDARD	
		**Positive**	**Negative**
**ALND software** TEST	**Positive**	56 TRUE POSITIVE	943 FALSE POSITIVE
	**Negative**	36 FALSE NEGATIVE	4687 TRUE NEGATIVE

***C**. accuracy of cancer detection by clinical reports*		**MDT-reported cancer** REFERENCE STANDARD	
		**Positive**	**Negative**
**Clinical report** TEST	**Positive**	61 TRUE POSITIVE	110 FALSE POSITIVE
	**Negative**	31 FALSE NEGATIVE	5520 TRUE NEGATIVE

***D**. accuracy of cancer detection by software, where abnormality was visible on radiograph*		**MDT-reported cancer** REFERENCE STANDARD	
		**Positive**	**Negative**
**Clinical report** TEST	**Positive**	51 TRUE POSITIVE	943 FALSE POSITIVE
	**Negative**	24 FALSE NEGATIVE	4687 TRUE NEGATIVE

***E**. accuracy of cancer detection by clinical reports, where abnormality was visible on radiograph*		**MDT-reported cancer** REFERENCE STANDARD	
		**Positive**	**Negative**
**Clinical report** TEST	**Positive**	60 TRUE POSITIVE	110 FALSE POSITIVE
	**Negative**	15 FALSE NEGATIVE	5520 TRUE NEGATIVE

Supplementary Table 1. Contingency tables. **A**: performance of the software compared to clinical reports. **B**: performance of the software compared to the MDT diagnosis of cancer. **C**: performance of the clinical reports compared to the MDT diagnosis of cancer. **D**: subgroup analysis; performance of the software compared to MDT diagnosis of cancer, where the abnormality was visible on the radiograph. **E**: subgroup analysis; performance of the clinical reports compared to MDT diagnosis of cancer, where the abnormality was visible on the radiograph.

Test	Suspicious nodule identified by software	Any suspicious abnormality on clinical report*
**Reference standard**	Cancer diagnosis by MDT decision, where the abnormality was visible on the radiograph	
**Sensitivity**	68.0%<br>[56.2 - 78.3]	80.0%<br>[69.2 - 88.4]
**Specificity**	83.3%<br>[82.3 - 84.2]	98.0%<br>[97.7 - 98.4]
**PPV**	5.1%<br>[4.4 - 6.0]	35.3%<br>[30.5 - 40.4]
**NPV**	99.5%<br>[99.3 - 99.6]	99.7%<br>[99.6 - 99.8]
**Accuracy**	83.0%<br>[82.0 - 84.0]	97.8%<br>[97.4 - 98.2]
**FPPI**	0.18	0.02

Supplementary Table 2. Subgroup analysis. Performance of the software and clinical reports against MDT diagnosis of cancer, where the abnormality was visible on the radiograph, with 95% confidence intervals. PPV = positive predictive value, NPV = negative predictive value, FPPI = false positives per image. *Includes nodules, masses and secondary features of malignancy.

943 false positive results	
**Normal anatomy (including variants and age-related changes)**	**659 cases (69.9%)**
Musculoskeletal (e.g. first rib, transverse processes of spine)	52.8%
Vessels (e.g. composite shadowing)	8.5%
Normal hila	29.9%
Nipple	7.0%
Foreign body (e.g. pacemaker)	3.8%
Other (e.g. bowel, age-related changes)	5.2%
Kyphosis or scoliosis	3.0%
**Technical factors**	**45 cases (4.8%)**
Projectional artefact (e.g. rotation)	73.3%
Expiratory or suboptimal inspiration	31.1%
**Non-cancerous pathology**	**294 cases (31.2%)**
Infective changes (e.g. consolidation, atelectasis)	40.8%
Benign-appearing nodule (e.g. hamartoma, granuloma)	13.3%
Fluid overload or pulmonary oedema	4.4%
Pulmonary fibrosis or scarring	15.6%
Pleural plaques	19.0%
Bronchiectasis	3.7%
Rib fracture	9.5%
Other	4.4%

Supplementary Table 3. Failure analysis of the 943 false positive results yielded by the ALND software. Broad causes of failure included normal anatomy, technical factors and non-cancerous pathology. For each of these, the relative proportion of specific underlying causes is provided. Note that in some cases multiple factors were identified.

Section / Topic	No.	Item	
TITLE / ABSTRACT			
	**1**	Identification as a study of AI methodology, specifying the category of technology used (e.g., deep learning)	**Yes**
	**2**	Structured summary of study design, methods, results, and conclusions	**Yes**
INTRODUCTION			
	**3**	Scientific and clinical background, including the intended use and clinical role of the AI approach	**Yes**
	**4**	Study objectives and hypotheses	**Yes**
METHODS			
Study Design	**5**	Prospective or retrospective study	**Yes**
	**6**	Study goal, such as model creation, exploratory study, feasibility study, non-inferiority trial	**Yes**
Data	**7**	Data sources	**Yes**
	**8**	Eligibility criteria: how, where, and when potentially eligible participants or studies were identified (e.g., symptoms, results from previous tests, inclusion in registry, patient-care setting, location, dates)	**Yes**
	**9**	Data pre-processing steps	**Yes**
	**10**	Selection of data subsets, if applicable	**N/A**
	**11**	Definitions of data elements, with references to Common Data Elements	**N/A**
	**12**	De-identification methods	**N/A**
	**13**	How missing data were handled	**N/A**
Ground Truth	**14**	Definition of ground truth reference standard, in sufficient detail to allow replication	**Yes**
	**15**	Rationale for choosing the reference standard (if alternatives exist)	**Yes**
	**16**	Source of ground-truth annotations; qualifications and preparation of annotators	**N/A**
	**17**	Annotation tools	**N/A**
	**18**	Measurement of inter- and intrarater variability; methods to mitigate variability and/or resolve discrepancies	**N/A**
Data Partitions	**19**	Intended sample size and how it was determined	**N/A**
	**20**	How data were assigned to partitions; specify proportions	**N/A**
	**21**	Level at which partitions are disjoint (e.g., image, study, patient, institution)	**N/A**
Model	**22**	Detailed description of model, including inputs, outputs, all intermediate layers and connections	**N/A**
	**23**	Software libraries, frameworks, and packages	**N/A**
	**24**	Initialization of model parameters (e.g., randomization, transfer learning)	**N/A**
Training	**25**	Details of training approach, including data augmentation, hyperparameters, number of models trained	**N/A**
	**26**	Method of selecting the final model	**N/A**
	**27**	Ensembling techniques, if applicable	**N/A**
Evaluation	**28**	Metrics of model performance	**N/A**
	**29**	Statistical measures of significance and uncertainty (e.g., confidence intervals)	**N/A**
	**30**	Robustness or sensitivity analysis	**N/A**
	**31**	Methods for explainability or interpretability (e.g., saliency maps), and how they were validated	**N/A**
	**32**	Validation or testing on external data	**N/A**
RESULTS			
Data	**33**	Flow of participants or cases, using a diagram to indicate inclusion and exclusion	**Yes**
	**34**	Demographic and clinical characteristics of cases in each partition	**Yes**
Model performance	**35**	Performance metrics for optimal model(s) on all data partitions	**Yes**
	**36**	Estimates of diagnostic accuracy and their precision (such as 95% confidence intervals)	**Yes**
	**37**	Failure analysis of incorrectly classified cases	**Yes**
DISCUSSION			

	**38**	Study limitations, including potential bias, statistical uncertainty, and generalizability	**Yes**
	**39**	Implications for practice, including the intended use and/or clinical role	**Yes**
OTHER INFORMATION			
	**40**	Registration number and name of registry	**N/A**
	**41**	Where the full study protocol can be accessed	**N/A**
	**42**	Sources of funding and other support; role of funders	**Yes**

Supplementary Table 4. CLAIM checklist
