# Supplementary material for: Evaluating the performance of artificial intelligence software for lung nodule detection on chest radiographs in a retrospective real-world UK population
Source: BMJ Open. 2023 Nov 8;13(11):e077348. doi: 10.1136/bmjopen-2023-077348 (PMC10632826; doi:10.1136/bmjopen-2023-077348)
Supplement: Author's manuscript [file bmjopen-2023-077348.draft_revisions.pdf]

BMJ Open is committed to open peer review. As part of this commitment we make the peer review history of every article we publish publicly available.

When an article is published we post the peer reviewers' comments and the authors' responses online. We also post the versions of the paper that were used during peer review. These are the versions that the peer review comments apply to.

The versions of the paper that follow are the versions that were submitted during the peer review process. They are not the versions of record or the final published versions. They should not be cited or distributed as the published version of this manuscript.

BMJ Open is an open access journal and the full, final, typeset and author-corrected version of record of the manuscript is available on our site with no access controls, subscription charges or pay-per-view fees (http://bmjopen.bmj.com).

If you have any questions on BMJ Open's open peer review process please email info.bmjopen@bmj.com

BMJ Open

Artificial intelligence for lung nodule detection on chest radiographs: performance of commercially available software in a real-world UK population.

Journal:	*BMJ Open*
Manuscript ID	bmjopen-2023-077348
Article Type:	Original research
Date Submitted by the Author:	07-Jul-2023
Complete List of Authors:	Maiter, Ahmed; The University of Sheffield, Infection, Immunity and Cardiovascular Disease; Sheffield Teaching Hospitals NHS Foundation Trust, Radiology<br>Hocking, Katherine; Sheffield Teaching Hospitals NHS Foundation Trust, Radiology<br>Matthews, Suzanne; Sheffield Teaching Hospitals NHS Foundation Trust<br>Taylor, Jonathan; Sheffield Teaching Hospitals NHS Foundation Trust, 3D Lab<br>Sharkey, Michael; Sheffield Teaching Hospitals NHS Foundation Trust, 3D Lab<br>Metherall, Peter; Sheffield Teaching Hospitals NHS Foundation Trust, 3D Lab<br>Alabed, Samer; The University of Sheffield, Infection, Immunity and Cardiovascular Disease; Sheffield Teaching Hospitals NHS Foundation Trust, Radiology<br>Dwivedi, Krit; The University of Sheffield, Infection, Immunity and Cardiovascular Disease; Sheffield Teaching Hospitals NHS Foundation Trust, Radiology<br>Shahin, Yousef; The University of Sheffield, Infection, Immunity and Cardiovascular Disease; Sheffield Teaching Hospitals NHS Foundation Trust, Radiology<br>Anderson, Elizabeth; Sheffield Teaching Hospitals NHS Foundation Trust, Radiology<br>Holt, Sarah; Sheffield Teaching Hospitals NHS Foundation Trust, Radiology<br>Rowbotham, Charlotte; Sheffield Teaching Hospitals NHS Foundation Trust, Radiology<br>Kamil, Mohamed; Sheffield Teaching Hospitals NHS Foundation Trust, Radiology<br>Hoggard, N; The University of Sheffield, Infection, Immunity and Cardiovascular Disease; Sheffield Teaching Hospitals NHS Foundation Trust, Radiology<br>Balasubramanian, Saba; Sheffield Teaching Hospitals NHS Foundation Trust<br>Swift, Andrew; The University of Sheffield, Infection, Immunity and Cardiovascular Disease; Sheffield Teaching Hospitals NHS Foundation Trust, Radiology<br>Johns, Christopher; Sheffield Teaching Hospitals NHS Foundation Trust, Radiology

Keywords:	Diagnostic Imaging, Chest imaging < RADIOLOGY & IMAGING, Diagnostic radiology < RADIOLOGY & IMAGING

SCHOLARONE™
Manuscripts

I, the Submitting Author has the right to grant and does grant on behalf of all authors of the Work (as defined in the below author licence), an exclusive licence and/or a non-exclusive licence for contributions from authors who are: i) UK Crown employees; ii) where BMJ has agreed a CC-BY licence shall apply, and/or iii) in accordance with the terms applicable for US Federal Government officers or employees acting as part of their official duties; on a worldwide, perpetual, irrevocable, royalty-free basis to BMJ Publishing Group Ltd ("BMJ") its licensees and where the relevant Journal is co-owned by BMJ to the co-owners of the Journal, to publish the Work in this journal and any other BMJ products and to exploit all rights, as set out in our licence.

The Submitting Author accepts and understands that any supply made under these terms is made by BMJ to the Submitting Author unless you are acting as an employee on behalf of your employer or a postgraduate student of an affiliated institution which is paying any applicable article publishing charge ("APC") for Open Access articles. Where the Submitting Author wishes to make the Work available on an Open Access basis (and intends to pay the relevant APC), the terms of reuse of such Open Access shall be governed by a Creative Commons licence – details of these licences and which Creative Commons licence will apply to this Work are set out in our licence referred to above.

Other than as permitted in any relevant BMJ Author's Self Archiving Policies, I confirm this Work has not been accepted for publication elsewhere, is not being considered for publication elsewhere and does not duplicate material already published. I confirm all authors consent to publication of this Work and authorise the granting of this licence.

For peer review only

Artificial intelligence for lung nodule detection on chest radiographs: performance of commercially available software in a real-world UK population

For peer review only

For peer review only - http://bmjopen.bmj.com/site/about/guidelines.xhtml

Abstract

Objectives:

Early identification of lung cancer on chest radiographs improves patient outcomes. AI tools may increase diagnostic accuracy and streamline this pathway. This study evaluated the performance of commercially available AI-based software trained to identify cancerous lung nodules on chest radiographs.

Design:

This retrospective study included primary care chest radiographs acquired in a U.K. centre. The software evaluated each radiograph independently and outputs were compared with two reference standards: 1) the radiologist report and 2) the diagnosis of cancer by multidisciplinary team decision. Failure analysis was performed by interrogating the software marker locations on radiographs.

Participants:

5,722 consecutive chest radiographs were included from 5,592 patients (median age 59 years, 53.8% female, 1.6% prevalence of cancer).

Results:

Compared to radiologist reports for nodule detection, the software demonstrated sensitivity 54.5% [95%CI 44.2-64.4%], specificity 83.2% [82.2-84.1%], PPV 5.5% [4.6-6.6%] and NPV 99.0% [98.8-99.2%]. Compared to cancer diagnosis, the software demonstrated sensitivity 60.1% [50.1-70.1%], specificity 83.3% [82.3-84.2%], PPV 5.6% [4.8-6.6%] and NPV 99.2% [99.0-99.4%]. Normal or variant anatomy was misidentified as an abnormality in 69.9% of the 943 false positive cases.

Conclusions:

The software demonstrated considerable underperformance in this real-world patient cohort. Failure analysis suggested a lack of generalisability in the training and testing datasets as a potential factor. The low PPV carries the risk of over-investigation and limits the translation of the software to clinical practice. Our findings highlight the importance of training and testing software in representative datasets, with broader implications for the implementation of AI tools in imaging.

For peer review only

For peer review only - http://bmjopen.bmj.com/site/about/guidelines.xhtml

Keywords

Artificial intelligence; machine learning; computer-aided diagnosis; chest radiograph; lung nodule; lung cancer; diagnostic accuracy

Abbreviations

AI	Artificial intelligence
ALND	Auto Lung Nodule Detection
CI	Confidence interval
CLAIM	Checklist for Artificial Intelligence in Medical Imaging
CT	Computed tomography
FPPI	False positives per image
IQR	Interquartile range
MDT	Multidisciplinary team
NOLCP	National Optimal Lung Cancer Pathway
NPV	Negative predictive value
PACS	Picture Archiving Communications System
PPV	Positive predictive value
RIS	Radiology Information System
UK	United Kingdom

For peer review only

Summary box

What is already known on this topic:

- AI tools have been developed to aid the detection of abnormalities on chest radiographs, including cancerous lung nodules.
- The AI tool used in this study was previously tested on enriched datasets with a high proportion of cancerous lung nodules.

What this study adds:

- Testing the AI tool in a real-world dataset of consecutive chest radiographs resulted in considerable underperformance with a high number of false positives.
- The results demonstrate that performance in enriched datasets may not generalise to performance in real-world clinical populations.
- The findings highlight that training and testing AI tools in the populations for which they are intended is important for ensuring performance and enabling implementation into clinical practice.

For peer review only

For peer review only - http://bmjopen.bmj.com/site/about/guidelines.xhtml

Introduction

Early diagnosis of cancer is essential to improve prognosis.[1,2] Lung cancer is the commonest cause of cancer deaths in the United Kingdom (UK), with a 10-year survival rate of only 10%.[3] Early lung cancer may be apparent on chest radiographs as a lung nodule, defined as a focal opacity measuring <30 mm.[4] Although the causes of lung nodules are broad, the detection of a new nodule on a chest radiograph should raise suspicion for cancer and usually necessitates further investigation with CT.[5]

In the UK, the National Optimal Lung Cancer Pathway (NOLCP) was developed to expedite diagnosis and treatment to improve lung cancer survival. The pathway goal is to commence treatment within 49 days of referral for patients with a confirmed diagnosis.[6] According to the NOLCP, patients with chest radiograph abnormalities that are suspicious for lung cancer should undergo diagnostic CT, to be performed and reported within 72 hours. This poses a challenge for healthcare services, particularly in the context of increasing demand for medical imaging and staff shortages.[7] The time taken from radiograph acquisition to reporting can delay the pathway and may be problematic in centres with reporting backlogs. Furthermore, chest radiographs are performed in high volumes for a variety of indications, with only a small proportion raising concern for cancer.

The application of artificial intelligence (AI) methods to medical imaging is expanding rapidly and promises a number of benefits.[8–10] Automated evaluation of images could help to triage studies for earlier reporting by radiologists, focusing resource allocation to cases where timely reporting is likely to have the largest clinical benefit. Recent years have seen an increase in the number of studies presenting AI approaches to the evaluation of chest radiographs, either alone or in combination with radiologists. These have included tools to assist with the identification of acute abnormalities such as pneumonia or pneumothorax[11–14], tuberculosis screening[15–17], and identification of lung cancer.[18–20] Studies have also evaluated how AI-based categorisation of chest radiographs could help to streamline clinical workflows[21-22]. As the field advances and AI tools become commercially available, it is imperative that their performance in real-world populations and clinical settings is evaluated and understood.

The Auto Lung Nodule Detection software (ALND; Samsung Electronics, Suwon, South Korea) was developed to aid identification of lung nodules on digital chest radiographs. The software was Class IIa medical devices directive UKCA and CE-marked, FDA-approved, and commercially available at the time of our study. The design, training and validation of the software has been reported previously.[23,24] The software is based on a deep

For peer review only - http://bmjopen.bmj.com/site/about/guidelines.xhtml

convolutional neural network algorithm, trained using an enriched dataset of 17,210 radiographs (79·7% normal and 20·3% with cancerous lung nodules) from a single South Korean centre. The software was subsequently tested on 800 radiographs (25·0% normal and 75·0% with cancerous lung nodules) from four external datasets (from South Korea, Germany and the United States). The performance when used alone varied between the four enriched datasets, with a sensitivity range of 51·1% to 79·1% and false positives per image (FPPI) rate of 0·10 to 0·30. However, the software has not been tested in an unselected real-world cohort of patients attending for routine chest radiographs.

This study aimed to evaluate the performance of ALND at detecting lung nodules on chest radiographs seen in routine practice in a tertiary UK centre. Radiologist reports and lung cancer multidisciplinary team (MDT) decisions were used as the reference standards for lung nodule and lung cancer diagnosis.

For peer review only

For peer review only - http://bmjopen.bmj.com/site/about/guidelines.xhtml

Materials and methods

This retrospective study was approved by a local research ethics committee (17/YH/0142). The study flow is indicated in Figure 1. The study is presented in accordance with the Checklist for Artificial Intelligence in Medical Imaging (CLAIM; Supplementary Materials).[25] Data analysis was performed using Prism (GraphPad, San Diego, CA, USA) and R Studio using the Tidyverse and EpiR packages.

1. Case selection

Chest radiographs were identified retrospectively from the local radiology information system. Radiographs were eligible for inclusion if they met all of the following criteria: 1) requested by the patient's general practitioner, 2) performed on adult patients 3) between July 1 2020 and February 26 2021, and acquired 3) in the posterior-anterior projection, 4) on one of three GC85A digital radiography systems (Samsung Electronics, Suwon, South Korea) running the software tool (ALND v1·0, Samsung Electronics, Suwon, South Korea) 5) at the same tertiary centre (REDACTED).

2. Evaluation of clinical reports

For each included radiograph, the clinical report was assessed retrospectively for an opinion on the presence of a suspicious nodule or other features of cancer. This was performed manually given the broad variation in reporting styles and language. For example, reports could identify a suspicious nodule explicitly or implicitly (e.g. 'two-week wait referral is advised' or "urgent CT is recommended").

3. Software results

Included radiographs were assessed by the software on the digital radiography system at the time of acquisition. For each radiograph, the software outputs included the original DICOM-formatted image, a binary assessment for the presence of a suspicious lung nodule (i.e. 'yes' or 'no'), the number of detected abnormalities and a copy of the image with a marker on each detected abnormality (*not* a contour of the abnormality, but only an indication of location). The outputs were sent to a research-only Picture Archiving Communications System (PACS) and were not accessible for clinical use.

For peer review only - http://bmjopen.bmj.com/site/about/guidelines.xhtml

4. Accuracy of nodule detection

For each case, the software output was compared with the corresponding clinical report, which was used as the reference standard for the presence of a suspicious nodule. Sensitivity, specificity, positive predictive value (PPV), negative predictive value (NPV), overall accuracy and FPPI were calculated from contingency tables for all cases. FPPI was calculated as the number of false positive results divided by the number of radiographs.

5. Accuracy of cancer diagnosis

Records from the local lung cancer multidisciplinary team (MDT) meetings were reviewed from the date of the first included radiograph to six months after the last included radiograph. Cases with a diagnosis of cancer by lung MDT decision were identified. Diagnosis of lung cancer by MDT opinion was used as the reference standard, against which the software output and clinical report were compared for each case. Sensitivity, specificity, PPV, NPV, overall accuracy and FPPI were calculated from contingency tables. In those cases where a cancer was identified by the software, the output was interrogated to ensure that the identified abnormality was in the correct location; if the marker had been placed incorrectly by the software this was counted as a negative result. For each case with a diagnosis of cancer, the radiograph was re-reviewed by one consultant thoracic radiologist (REDACTED, 11 years of experience) to determine if the abnormality was visible; subgroup analysis was performed excluding cases in which the cancerous abnormality was not visible on the radiograph.

6. Failure analysis of incorrectly classified cases

Failure analysis was performed for false positive and false negative outputs by the software in reference to the presence or absence of MDT-confirmed cancer. For each of these cases, the software outputs were manually compared with the radiograph, clinical report and MDT opinion.

For peer review only - http://bmjopen.bmj.com/site/about/guidelines.xhtml

Results

1. Included radiographs and patients

A total of 5,722 chest radiographs were included from 1 July 2020 to 26 February 2021. These were acquired for 5,592 different patients, with 2·3% of these having had either two or three radiographs. The included patients had a median age of 59 years (IQR 46-72 years), with 53·8% female (Figure 2A). 79·0% of patients were white or white British. Ethnicity information was unavailable for 9·1% of cases (Figure 2B). No radiographs or patients were excluded.

2. Clinical reports

All radiographs had been reported independently by FRCR-qualified radiologists, with 91·0% reported by 7 different consultant thoracic radiologists. 58·5% of reports mentioned comparison with previous imaging studies. Potentially cancerous abnormalities were identified in 3·0% of reports; these included nodules (59·1%), masses (25·7%) and other abnormalities such as lung volume loss or pleural thickening (21·6%).

3. Accuracy of nodule detection

All 5,722 chest radiographs were evaluated by the software, with none failing evaluation. The software identified a total of 1,120 potentially cancerous nodules on 17·5% of radiographs. When compared against identification of suspicious nodules in the clinical report (i.e. excluding masses or other abnormalities for which the software had not been trained), the software yielded a sensitivity of 54·5%, specificity of 83·2%, PPV of 5·5%, NPV of 99·0% and overall accuracy of 82·7%, with a FPPI of 0·18 (Table 1 and Supplementary Table 1A).

For peer review only

**Test**	Suspicious nodule identified by software	Suspicious nodule identified by software	Any suspicious abnormality on clinical report**
**Reference standard**	Suspicious nodule on clinical report*	Cancer diagnosis by MDT decision	
**Sensitivity**	54·5% [44·2 – 64·4]	60·9% [50·1 – 70·9]	66·3% [55·7 – 75·8]
**Specificity**	83·2% [82·2 – 84·1]	83·3% [82·3 – 84·2]	98·0% [97·7 – 98·4]
**PPV**	5·5% [4·6 – 6·6]	5·6% [4·8 – 6·6]	35·7% [30·5 – 41·2]
**NPV**	99·0% [98·8 – 99·2]	99·2% [99·0 – 99·4]	99·4% [99·3 – 99·6]
**Accuracy**	82·7% [81·7 – 83·6]	82·9% [81·9 – 83·9]	97·5% [97·1 – 97·9]
**FPPI**	0·18	0·18	0·02

Table 1. Performance of the software and clinical reports against reference standards, with 95% confidence intervals. PPV = positive predictive value, NPV = negative predictive value, FPPI = false positives per image. *Other suspicious abnormalities (including masses and secondary features of cancer, such as lung volume loss) were excluded. **Includes nodules, masses, and secondary features of malignancy (such as unilateral pleural effusions or lobar collapse).

4. Accuracy of cancer diagnosis

92 patients (1·6%) were found to have a diagnosis of cancer by MDT opinion. When the suspicious nodules identified by the software were compared against MDT diagnosis, the software yielded a sensitivity of 60·9%, specificity of 83·3%, PPV of 5·6%, NPV of 99·2% and accuracy of 82·9%, with a FPPI of 0·18 (Table 1 and Supplementary Table 1B). Examples of correct identification of cancer by the software are shown in Figure 3. When all suspicious abnormalities identified in the clinical reports (i.e. including nodules, masses and other abnormalities) were compared against MDT diagnosis, the clinical reports yielded a sensitivity of 66·3%, specificity of 98·0%, PPV of 35·7%, NPV of 99·4% and accuracy of 97·5%, with a FFPI of 0·02 (Table 1 and

For peer review only - http://bmjopen.bmj.com/site/about/guidelines.xhtml

Supplementary Table 1C). Hypothetically, if the detection of an abnormality automatically resulted in patients undergoing CT, the software would have resulted in 999 scans with 60·9% of cancer cases detected (Supplementary Table 1B). By comparison, the clinical reports would have resulted in 171 scans with 66·3% of cancer cases detected. (Supplementary Table 1C).

Of the 92 cases of cancer, the abnormality was deemed to be visible on the radiograph in 75 cases. Using these cases as the reference standard, the software yielded a sensitivity of 68·0%, specificity of 83·3%, PPV of 5·1%, NPV of 99·5% and accuracy of 83·0% (Supplementary Table 1D and Supplementary Table 2). The clinical reports yielded a sensitivity of 80·0%, specificity of 98·0%, PPV of 35·3%, NPV of 99·7% and accuracy of 97·8%. (Supplementary Table 1E and Supplementary Table 2). Performance of the clinical reports was similar to expected.[26]

5. Failure analysis of ALND results

The software yielded 34 false negatives compared to MDT opinion (36·9% of cases with cancer). In 22 of these cases, the abnormalities were found to be visible on the radiograph and included masses (63·5 %, defined as >30 mm), lung collapse secondary to obstructing cancer (18·2 %), solitary or multiple cancerous nodules (13·6%), malignant pleural effusion (9·1%) and persistent consolidation (9·1%) (Figure 4).

The software yielded 943 false positive results in comparison with MDT opinion, representing 16·8% of all cases without cancer. Only 7·8% of these cases had been reported as abnormal in the formal clinical report and were subsequently found to be due to a benign cause. Contrastingly, the formal clinical report yielded 110 false positive results compared to MDT opinion, representing 2·0% of cases without cancer. Causes of failure by the software included normal anatomy (69·9%), non-cancerous pathology (31·2%) and technical factors (4·8%) (Figure 5 and Supplementary Table 3).

For peer review only - http://bmjopen.bmj.com/site/about/guidelines.xhtml

Discussion

The evaluation of AI tools in real-world settings is essential for their translation to clinical practice. In this retrospective study, we evaluated the performance of an AI-based software tool trained to identify suspicious lung nodules on chest radiographs. The software demonstrated underperformance on consecutive patients referred from primary care. While sensitivity and FFPI values were similar to those reported previously for enriched datasets, we have demonstrated a considerably low PPV when tested in a real-world dataset. The results carry important implications for the training, evaluation, and clinical implementation of AI tools in medical imaging.

A large number of consecutive cases referred from primary care were included using broad inclusion criteria. We believe that this unenriched dataset is more representative of the population encountered in routine clinical practice. Our results showed sensitivity and FPPI values consistent with those reported during testing of the software.[23,24] However, we found low PPV values, which severely limit the potential utility of the software and make it unsuitable for our population. Compared to radiologist reports, the software underperformed in all measures of diagnostic performance. Our results raise important questions about the intended use of the software and how it could fit into a clinical pathway. The low PPV demonstrated here suggests that use of the software for its intended purpose would result in over-investigation of patients without an improvement in sensitivity. In comparison with the clinical reports, if all cases where the software had detected an abnormality suspicious for cancer had undergone CT, this would have resulted in 5.8 times the number of scans performed and fewer cases of cancer detected. Such over-investigation is harmful: unnecessary diagnostic tests carry physical risks such as increased doses of ionising radiation, may contribute to patient anxiety and distress, and can increase the demands on healthcare systems.

Understanding *why* underperformance has occurred is essential for further development of AI tools. As part of the output for each radiograph, the software labelled detected abnormalities. This is an important design feature, enabling failure analysis, allowing proper evaluation of performance, and aiding decision-making if used in clinical settings. The software frequently misidentified normal, variant, or age-related anatomy as abnormalities. This included false positives that radiologists readily dismiss, such as the ends of the first ribs. What factors could have contributed to this? One potential explanation is the use of insufficiently representative and generalisable datasets for software training and testing. The training and testing datasets included a prevalence of cancer far higher than that seen in real world clinical populations, which may have skewed performance towards overcalling abnormalities.[23] In contrast, our dataset had a more realistic prevalence of 1·6%. Differences in population

For peer review only - http://bmjopen.bmj.com/site/about/guidelines.xhtml

demographics may have also contributed to underperformance. The software was trained on a South Korean population.[23] Gichoya et al.[27,28] recently demonstrated that AI tools are capable of determining a patient's race from radiographs, highlighting that there can be an inherent difference in normal imaging appearances between populations. An AI model trained on data from one population may not generalise well to others that show significant demographic differences. The interaction of AI with patient factors such as sex, race, and ethnicity is an important practical and ethical issue for the field moving forwards. Alternatively, insufficient variation and volume in the 'normal' radiographs used in training may have affected performance. The spectrum of normality is broad and can encompass variations in anatomy and technical factors relating to radiograph acquisition. How 'normal' radiographs were defined in the training and testing datasets was not reported clearly. For example, did these include anatomical variants, age-related changes, radiographs that were rotated, or artefacts from external clothing - all of which are seen commonly in routine practice? And if so, were these included in a sufficient and representative number? Furthermore, the training and testing datasets did not include non-cancerous pathology. For example, smoking and industrial exposures are risk factors for lung cancer and cause benign abnormalities that are visible on chest radiographs, such as emphysema or pleural plaques. It is important for an AI tool to be able to identify cancer in a population of patients with co-existing pathologies that reflect an increased cancer risk. Masses and secondary features of cancer (such as pleural effusion or mediastinal lymphadenopathy) were also absent from the training and testing datasets. Finally, the software was trained to assess radiographs in isolation, with no comparison to previous imaging or clinical context, in stark contrast to how radiologists interpret imaging. In short, training and testing datasets need to be representative of the intended population to ensure generalisability of AI tools, and models should ideally integrate information from previous imaging studies, patient demographics and clinical history. While existing studies have often used enriched data sets to develop and test models, promising models should be tested in representative real-world datasets and environments prior to their routine clinical use.

Our study has limitations. The retrospective design allowed inclusion of a large number of cases but does not afford the same degree of robustness or control over confounding variables as a prospective study. Using data from routine clinical care risks incomplete information, such as a lack of documented ethnicity in some patients. There is also inherent difficulty in defining the reference standard for a suspicious nodule. Other studies have used CT or biopsy results, but such approaches are less practical when evaluating a large real-world clinical cohort and introduce their own selection bias: some inflammatory nodules will be transient and not warrant further investigation, some patients may not undergo CT or biopsy, or some results may be indeterminate. We used

For peer review only - http://bmjopen.bmj.com/site/about/guidelines.xhtml

cancers diagnosed by lung MDT decision as a reference standard, but some cases of cancer may have been missed if they had not been referred to or reviewed by the MDT, or if they represented metastatic disease to the lung from another known primary cancer. Finally, assessment of the clinical reports and failure analysis were performed by radiologists, with the risk of interobserver variability or observer bias. Regardless, we believe that the study limitations are unlikely to explain the considerable differences in performance compared to the software's prior validation and testing. Looking ahead, it is important that software tools showing promising performance in representative datasets such as ours are then more formally evaluated prospectively, such as through randomised controlled trials.

The excitement surrounding AI is understandable given the promise of automation in an era of growing demands for medical imaging. The Royal College of Radiologists reports a shortfall of 29% of the UK radiologist workforce, raising concerns about reporting backlogs and the potential for delayed diagnoses.[7] Chest radiographs remain a mainstay of imaging in radiology departments, with a median of just under 650,000 performed per month in England in 2021-2022.[29] The ability to streamline reporting would assist in dealing with this high burden, addressing backlogs and minimising the impacts of service pressures. Early identification of patients with possible lung cancer on chest radiographs would facilitate more efficient workflows, reducing the time to diagnosis and improving patient outcomes.[1,2] However, this study highlights fundamental questions about the application of AI in medical imaging. Firstly, what constitutes sufficient training and testing? We have demonstrated that performance in narrow and enriched datasets during software development is not necessarily generalisable to real-world populations. This issue is likely to become more significant as the field advances and model performances improve.[21] However, when the pre-test probability of the disease is low, large population samples will be required to demonstrate significant differences in performance. There remain significant ethical concerns about the systematic propagation of human bias in AI tools that may disadvantage minority populations[27,28,30,31] and datasets need to be selected carefully and presented in a transparent manner. Secondly, what are the appropriate benchmarks for performance? In this study, we have not only compared the software performance against radiologist reports, but also MDT diagnosis of lung cancer, which accounted for clinical, imaging, and histopathological factors. Proper definition of reference standards can be challenging, particularly in clinical settings. Thirdly, how should performance be presented and interpreted? High-quality reporting of model design, training, validation, and testing is crucial for understanding the performance and generalisability of AI tools.[32] Transparency of AI tool development and performance is essential for the gaining the trust of stakeholders, including the public, and is therefore important for translation of tools into the clinical sphere. The way AI tools

For peer review only - http://bmjopen.bmj.com/site/about/guidelines.xhtml

are presented should be consistent, enabling direct comparisons of performance, and accessible to all stakeholders, allowing purpose and performance to be understood by those without extensive experience in the field of AI. Poor performance should also be presented, analysed, and published in a fair manner to minimise bias within the field. Lastly, where do we see such AI tools fitting into clinical pathways? We found a low PPV, which combined with a low prevalence of disease in the studied population, suggest that implementation of the software would result in over-investigation and potentially *contributing* to the burden on healthcare systems. Conversely, the NPV of the software exceeded 99%, and there could be potential utility in using the software to streamline reporting by removing studies that do not have cancer. Other studies have presented the use of AI approaches to categorise chest radiographs for the purpose of report prioritisation[21,22], which is of particular appeal given the large volumes requiring reporting. However, in this case the software would also exclude non-cancerous abnormalities that could still be significant (such as infection) and would offer no improvement over a radiologist's NPV, and therefore may not add much clinical value. Alternatively, AI tools may be of benefit when used to augment reporting by radiologists or to provide a second reading.[13,33,34] In order to streamline workflows and pathways, performance thresholds need to be chosen carefully and determined prior to software development.

Conclusion

Evaluation of AI-based software tools in representative patient cohorts is crucial for their translation to routine clinical practice. We have demonstrated underperformance of the software using a large retrospective and relatively unselected clinical cohort, despite apparent similar diagnostic accuracy in comparison to the initial training and testing. Understanding the external validity of software performance is an important consideration as the field continues to advance.

For peer review only - http://bmjopen.bmj.com/site/about/guidelines.xhtml

References

1 Yang C-FJ, Wang H, Kumar A, *et al.* Impact of Timing of Lobectomy on Survival for Clinical Stage IA Lung Squamous Cell Carcinoma. *Chest* 2017; **152**: 1239–50.

2 Navani N, Nankivell M, Lawrence DR, *et al.* Lung cancer diagnosis and staging with endobronchial ultrasound-guided transbronchial needle aspiration compared with conventional approaches: an open-label, pragmatic, randomised controlled trial. *Lancet Respir Med* 2015; **3**: 282–9.

3 Survival | Lung cancer | Cancer Research UK. https://www.cancerresearchuk.org/about-cancer/lung-cancer/survival (accessed Dec 10, 2022).

4 Quadrelli S, Lyons G, Colt H, Chimondeguy D, Buero A. Clinical Characteristics and Prognosis of Incidentally Detected Lung Cancers. *Int J Surg Oncol* 2015; **2015**: e287604.

5 National Institute for Health and Care Excellence (NICE). Lung cancer: diagnosis and management. https://www.nice.org.uk/guidance/ng122 (accessed Dec 10, 2022).

6 NHS England. Rapid cancer diagnostic and assessment pathways. https://www.england.nhs.uk/publication/rapid-cancer-diagnostic-and-assessment-pathways/ (accessed Dec 10, 2022).

7 The Royal College of Radiologists. Clinical radiology census report 2021. 2022. https://www.rcr.ac.uk/clinical-radiology/rcr-clinical-radiology-census-report-2021 (accessed Jan 5, 2023).

8 Hosny A, Parmar C, Quackenbush J, Schwartz LH, Aerts HJWL. Artificial intelligence in radiology. *Nat Rev Cancer* 2018; **18**: 500–10.

9 Killock D. AI outperforms radiologists in mammographic screening. *Nat Rev Clin Oncol* 2020; **17**: 134–134.

10 Neri E, de Souza N, Brady A, *et al.* What the radiologist should know about artificial intelligence – an ESR white paper. *Insights Imaging* 2019; **10**: 44.

11 Hwang EJ, Park S, Jin K-N, *et al.* Development and Validation of a Deep Learning–Based Automated Detection Algorithm for Major Thoracic Diseases on Chest Radiographs. *JAMA Netw Open* 2019; **2**: e191095.

12 Singh R, Kalra MK, Nitiwarangkul C, *et al.* Deep learning in chest radiography: Detection of findings and presence of change. *PloS One* 2018; **13**: e0204155.

13 Choi SY, Park S, Kim M, Park J, Choi YR, Jin KN. Evaluation of a deep learning-based computer-aided detection algorithm on chest radiographs: Case-control study. *Medicine (Baltimore)* 2021; **100**: e25663.

14 Nam JG, Kim M, Park J, *et al.* Development and validation of a deep learning algorithm detecting 10 common abnormalities on chest radiographs. *Eur Respir J* 2021; **57**. DOI:10.1183/13993003.03061-2020.

15 Codlin AJ, Dao TP, Vo LNQ, *et al.* Independent evaluation of 12 artificial intelligence solutions for the detection of tuberculosis. *Sci Rep* 2021; **11**: 23895.

16 Lee JH, Park S, Hwang EJ, *et al.* Deep learning-based automated detection algorithm for active pulmonary tuberculosis on chest radiographs: diagnostic performance in systematic screening of asymptomatic individuals. *Eur Radiol* 2021; **31**: 1069–80.

17 Nash M, Kadavigere R, Andrade J, *et al.* Deep learning, computer-aided radiography reading for tuberculosis: a diagnostic accuracy study from a tertiary hospital in India. *Sci Rep* 2020; **10**: 210.

18 Yoo H, Kim KH, Singh R, Digumarthy SR, Kalra MK. Validation of a Deep Learning Algorithm for the Detection of Malignant Pulmonary Nodules in Chest Radiographs. *JAMA Netw Open* 2020; **3**: e2017135.

For peer review only - http://bmjopen.bmj.com/site/about/guidelines.xhtml

19 Koo YH, Shin KE, Park JS, Lee JW, Byun S, Lee H. Extravalidation and reproducibility results of a commercial deep learning-based automatic detection algorithm for pulmonary nodules on chest radiographs at tertiary hospital. *J Med Imaging Radiat Oncol* 2021; **65**: 15–22.

20 Nam JG, Park S, Hwang EJ, *et al.* Development and Validation of Deep Learning–based Automatic Detection Algorithm for Malignant Pulmonary Nodules on Chest Radiographs. *Radiology* 2019; **290**: 218–28.

21 Dyer T, Dillard L, Harrison M, *et al.* Diagnosis of normal chest radiographs using an autonomous deep-learning algorithm. *Clin Radiol* 2021; **76**: 473.e9-473.e15.

22 Annarumma M, Withey SJ, Bakewell RJ, Pesce E, Goh V, Montana G. Automated Triaging of Adult Chest Radiographs with Deep Artificial Neural Networks. *Radiology* 2019; **291**: 196–202.

23 Sim Y, Chung MJ, Kotter E, *et al.* Deep Convolutional Neural Network–based Software Improves Radiologist Detection of Malignant Lung Nodules on Chest Radiographs. *Radiology* 2020; **294**: 199–209.

24 Jacobson FL. Medical Image Perception Research in the Emerging Age of Artificial Intelligence. *Radiology* 2020; **294**: 210–1.

25 Mongan J, Moy L, Kahn CE. Checklist for Artificial Intelligence in Medical Imaging (CLAIM): A Guide for Authors and Reviewers. *Radiol Artif Intell* 2020; **2**: e200029.

26 Aberle DR, DeMello S, Berg CD, *et al.* Results of the Two Incidence Screenings in the National Lung Screening Trial. *N Engl J Med* 2013; **369**: 920–31.

27 Gichoya JW, Banerjee I, Bhimireddy AR, *et al.* AI recognition of patient race in medical imaging: a modelling study. *Lancet Digit Health* 2022; **4**: e406–14.

28 Wiens J, Creary M, Sjoding MW. AI models in health care are not colour blind and we should not be either. *Lancet Digit Health* 2022; **4**: e399–400.

29 NHS England. Diagnostic Imaging Dataset 2021-22 Data. https://www.england.nhs.uk/statistics/statistical-work-areas/diagnostic-imaging-dataset/diagnostic-imaging-dataset-2021-22-data/ (accessed Jan 5, 2023).

30 Seyyed-Kalantari L, Zhang H, McDermott MBA, Chen IY, Ghassemi M. Underdiagnosis bias of artificial intelligence algorithms applied to chest radiographs in under-served patient populations. *Nat Med* 2021; **27**: 2176–82.

31 Char DS, Shah NH, Magnus D. Implementing Machine Learning in Health Care — Addressing Ethical Challenges. *N Engl J Med* 2018; **378**: 981–3.

32 Alabed S, Maiter A, Salehi M, *et al.* Quality of reporting in AI cardiac MRI segmentation studies – A systematic review and recommendations for future studies. *Front Cardiovasc Med* 2022; **9**. https://www.frontiersin.org/articles/10.3389/fcvm.2022.956811 (accessed Jan 6, 2023).

33 Tam MDBS, Dyer T, Dissez G, *et al.* Augmenting lung cancer diagnosis on chest radiographs: positioning artificial intelligence to improve radiologist performance. *Clin Radiol* 2021; **76**: 607–14.

34 McKinney SM, Sieniek M, Godbole V, *et al.* International evaluation of an AI system for breast cancer screening. *Nature* 2020; **577**: 89–94.

For peer review only

For peer review only - http://bmjopen.bmj.com/site/about/guidelines.xhtml

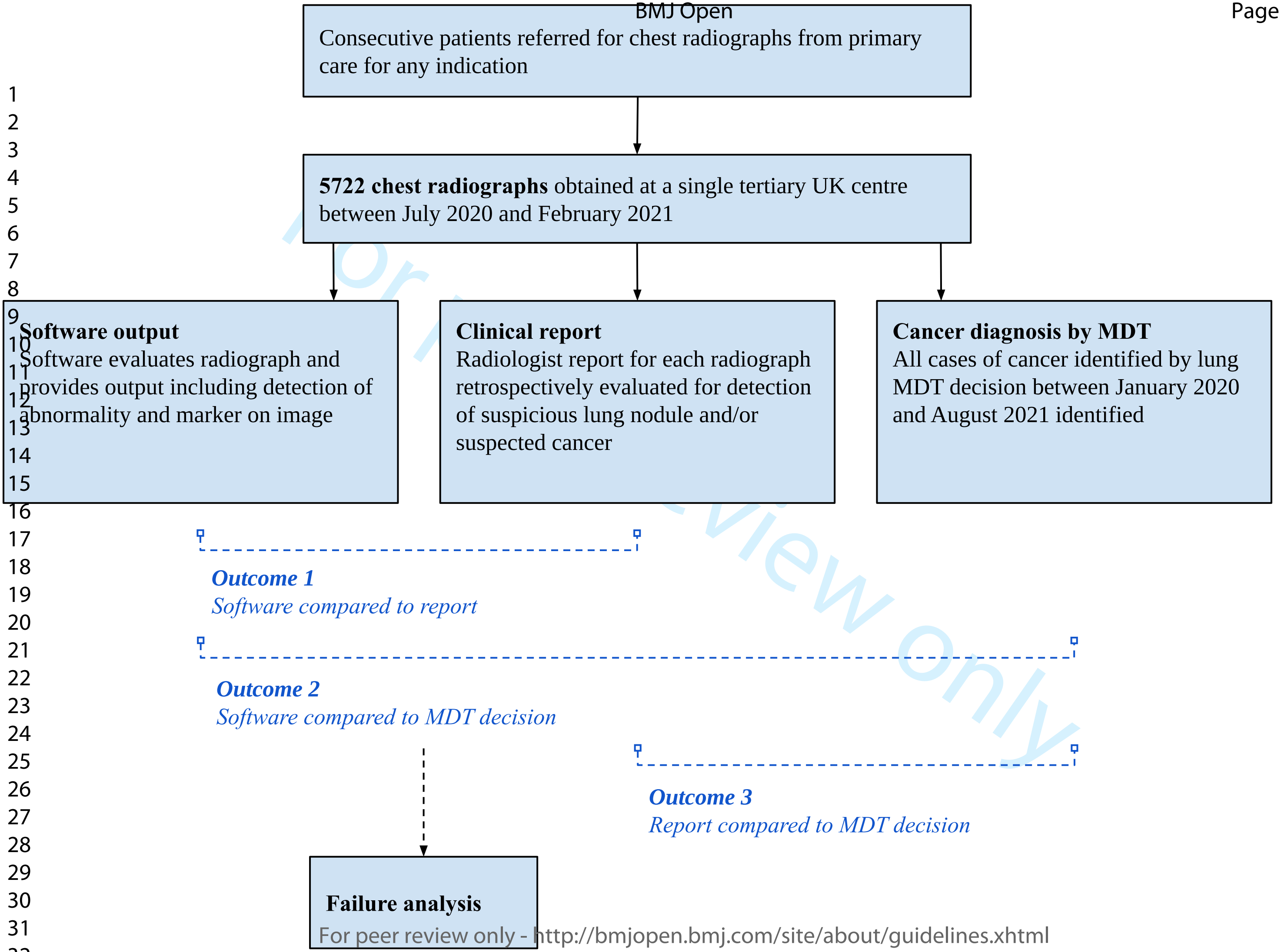

For peer review only - http://bmjopen.bmj.com/site/about/guidelines.xhtml

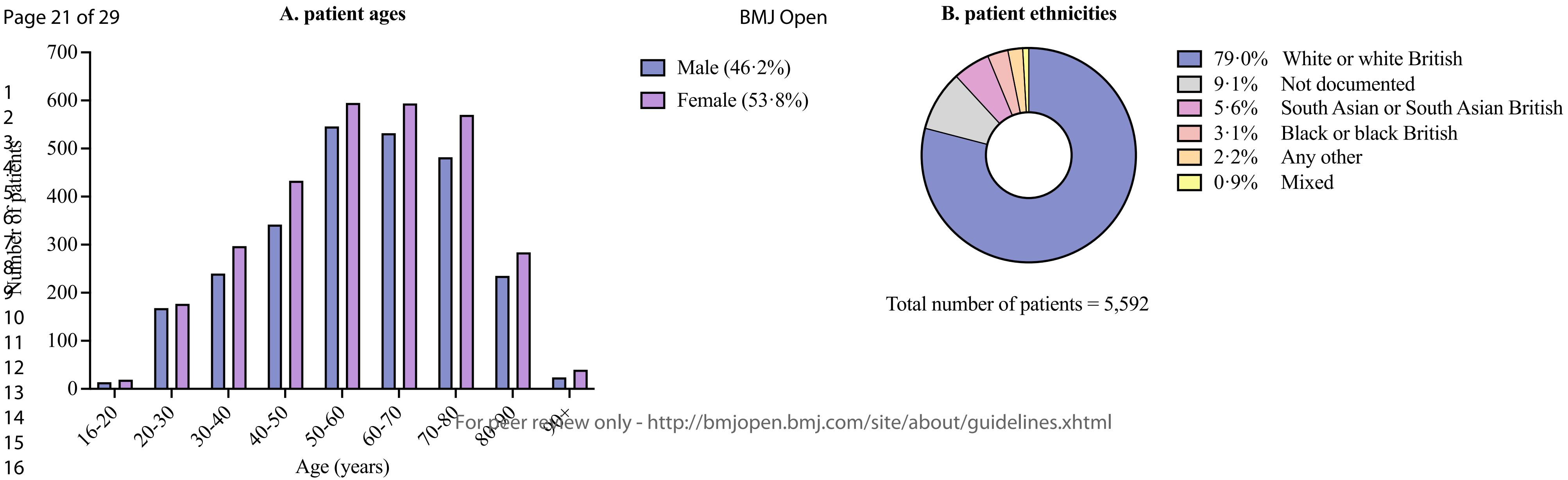

For peer review only - http://bmjopen.bmj.com/site/about/guidelines.xhtml

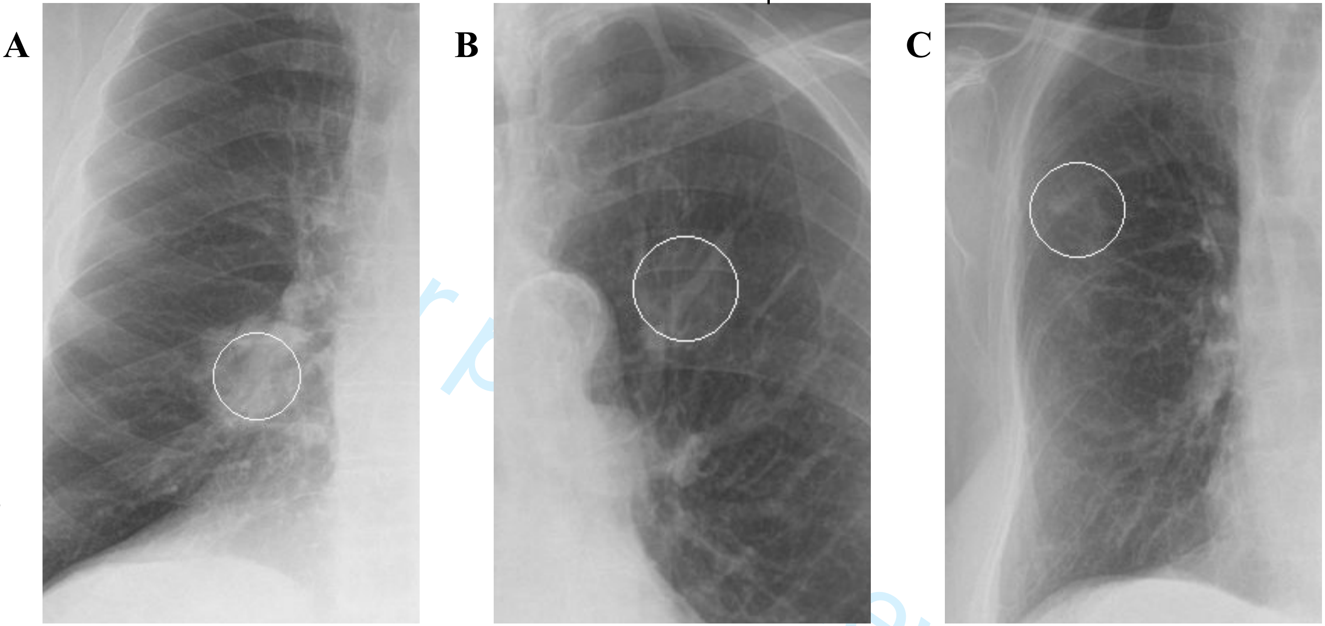

For peer review only - http://bmjopen.bmj.com/site/about/guidelines.xhtml

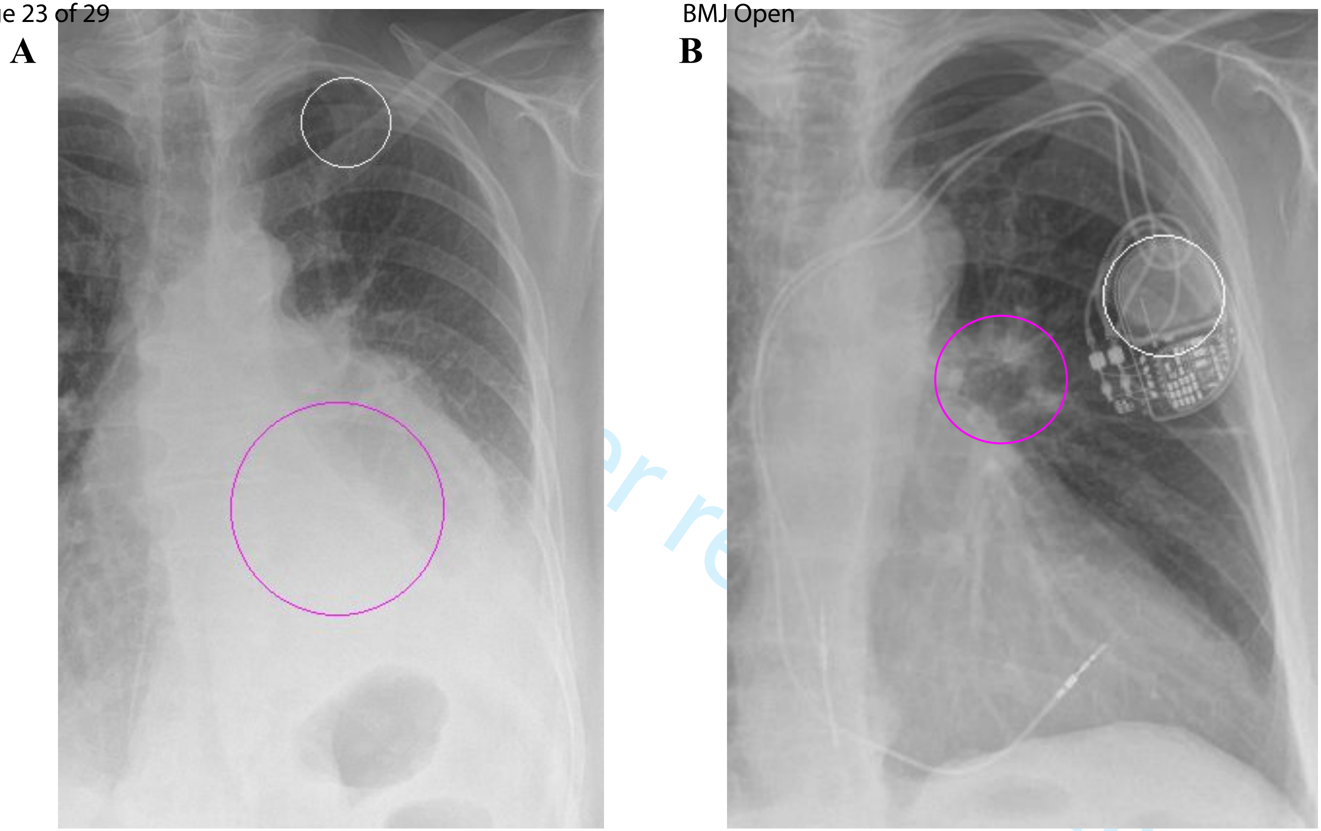
A
B

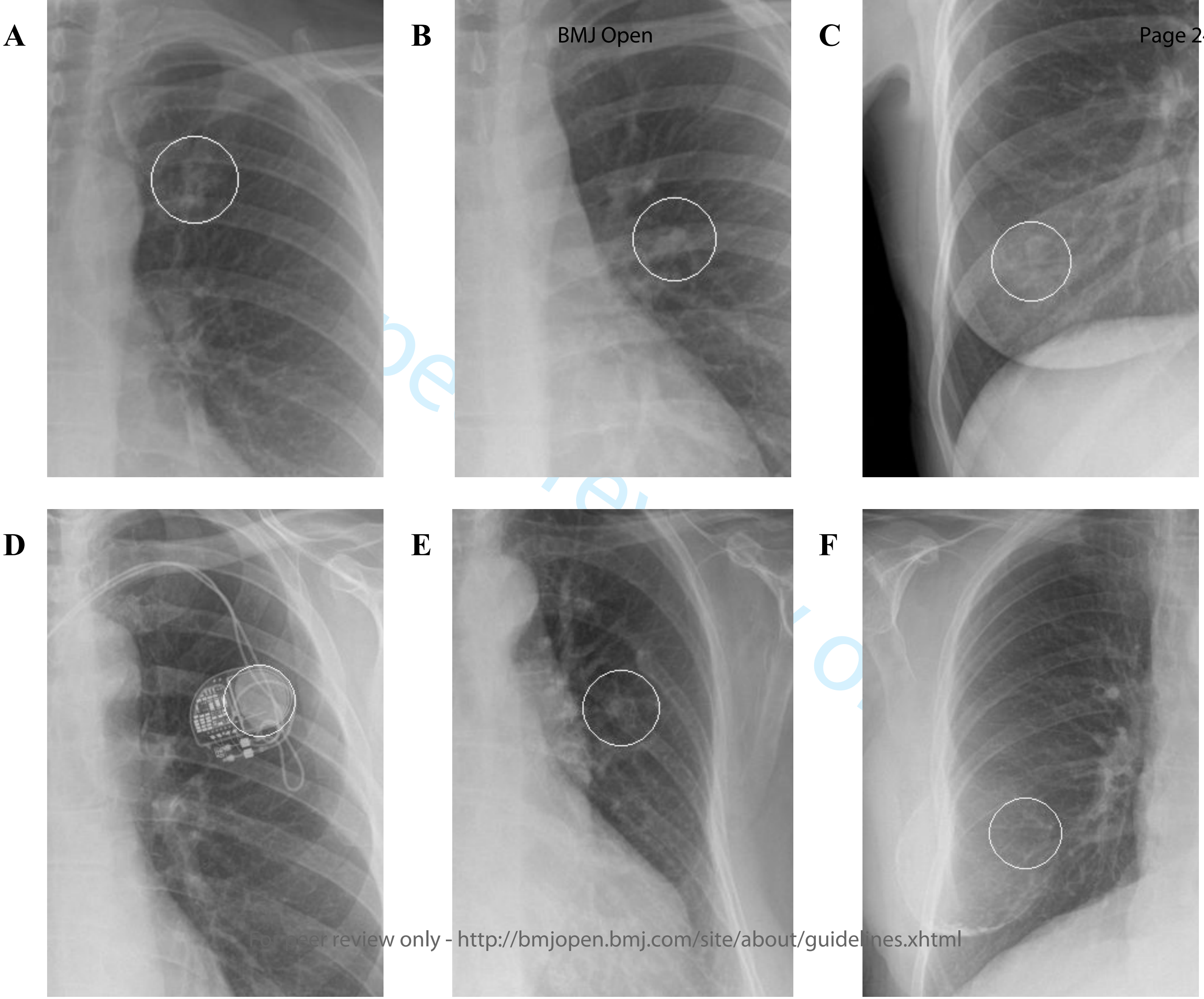

For peer review only - http://bmjopen.bmj.com/site/about/guidelines.xhtml

***A**. accuracy of nodule detection by software*		**Clinical report** REFERENCE STANDARD	
		**Positive**	**Negative**
**ALND software** TEST	**Positive**	55 TRUE POSITIVE	946 FALSE POSITIVE
	**Negative**	45 FALSE NEGATIVE	4676 TRUE NEGATIVE

***B**. accuracy of cancer detection by software*		**MDT-reported cancer** REFERENCE STANDARD	
		**Positive**	**Negative**
**ALND software** TEST	**Positive**	56 TRUE POSITIVE	943 FALSE POSITIVE
	**Negative**	36 FALSE NEGATIVE	4687 TRUE NEGATIVE

***C**. accuracy of cancer detection by clinical reports*		**MDT-reported cancer** REFERENCE STANDARD	
		**Positive**	**Negative**
**Clinical report** TEST	**Positive**	61 TRUE POSITIVE	110 FALSE POSITIVE
	**Negative**	31 FALSE NEGATIVE	5520 TRUE NEGATIVE

***D**. accuracy of cancer detection by software, where abnormality was visible on radiograph*		**MDT-reported cancer** REFERENCE STANDARD	
		**Positive**	**Negative**
**Clinical report** TEST	**Positive**	51 TRUE POSITIVE	943 FALSE POSITIVE
	**Negative**	24 FALSE NEGATIVE	4687 TRUE NEGATIVE

***E**. accuracy of cancer detection by clinical reports, where abnormality was visible on radiograph*		**MDT-reported cancer** REFERENCE STANDARD	
		**Positive**	**Negative**
**Clinical report** TEST	**Positive**	60 TRUE POSITIVE	110 FALSE POSITIVE
	**Negative**	15 FALSE NEGATIVE	5520 TRUE NEGATIVE

For peer review only

Supplementary Table 1. Contingency tables. **A**: performance of the software compared to clinical reports. **B**: performance of the software compared to the MDT diagnosis of cancer. **C**: performance of the clinical reports compared to the MDT diagnosis of cancer. **D**: subgroup analysis; performance of the software compared to MDT diagnosis of cancer, where the abnormality was visible on the radiograph. **E**: subgroup analysis; performance of the clinical reports compared to MDT diagnosis of cancer, where the abnormality was visible on the radiograph.

For peer review only

**Test**	Suspicious nodule identified by software	Any suspicious abnormality on clinical report*
**Reference standard**	Cancer diagnosis by MDT decision, where the abnormality was visible on the radiograph	
**Sensitivity**	68.0%<br>[56.2 - 78.3]	80.0%<br>[69.2 - 88.4]
**Specificity**	83.3%<br>[82.3 - 84.2]	98.0%<br>[97.7 - 98.4]
**PPV**	5.1%<br>[4.4 - 6.0]	35.3%<br>[30.5 - 40.4]
**NPV**	99.5%<br>[99.3 - 99.6]	99.7%<br>[99.6 - 99.8]
**Accuracy**	83.0%<br>[82.0 - 84.0]	97.8%<br>[97.4 - 98.2]
**FPPI**	0.18	0.02

Supplementary Table 2. Subgroup analysis. Performance of the software and clinical reports against MDT diagnosis of cancer, where the abnormality was visible on the radiograph, with 95% confidence intervals. PPV = positive predictive value, NPV = negative predictive value, FPPI = false positives per image. *Includes nodules, masses and secondary features of malignancy.

For peer review only

**943 false positive results**	
**Normal anatomy (including variants and age-related changes)**	**659 cases (69.9%)**
Musculoskeletal (e.g. first rib, transverse processes of spine)	52.8%
Vessels (e.g. composite shadowing)	8.5%
Normal hila	29.9%
Nipple	7.0%
Foreign body (e.g. pacemaker)	3.8%
Other (e.g. bowel, age-related changes)	5.2%
Kyphosis or scoliosis	3.0%
**Technical factors**	**45 cases (4.8%)**
Projectional artefact (e.g. rotation)	73.3%
Expiratory or suboptimal inspiration	31.1%
**Non-cancerous pathology**	**294 cases (31.2%)**
Infective changes (e.g. consolidation, atelectasis)	40.8%
Benign-appearing nodule (e.g. hamartoma, granuloma)	13.3%
Fluid overload or pulmonary oedema	4.4%
Pulmonary fibrosis or scarring	15.6%
Pleural plaques	19.0%
Bronchiectasis	3.7%
Rib fracture	9.5%
Other	4.4%

Supplementary Table 3. Failure analysis of the 943 false positive results yielded by the ALND software. Broad causes of failure included normal anatomy, technical factors and non-cancerous pathology. For each of these, the relative proportion of specific underlying causes is provided. Note that in some cases multiple factors were identified.

For peer review only - http://bmjopen.bmj.com/site/about/guidelines.xhtml

Section / Topic	No.	Item	
TITLE / ABSTRACT			
	1	Identification as a study of AI methodology, specifying the category of technology used (e.g., deep learning)	Yes
	2	Structured summary of study design, methods, results, and conclusions	Yes
INTRODUCTION			
	3	Scientific and clinical background, including the intended use and clinical role of the AI approach	Yes
	4	Study objectives and hypotheses	Yes
METHODS			
Study Design	5	Prospective or retrospective study	Yes
	6	Study goal, such as model creation, exploratory study, feasibility study, non-inferiority trial	Yes
Data	7	Data sources	Yes
	8	Eligibility criteria: how, where, and when potentially eligible participants or studies were identified (e.g., symptoms, results from previous tests, inclusion in registry, patient-care setting, location, dates)	Yes
	9	Data pre-processing steps	Yes
	10	Selection of data subsets, if applicable	N/A
	11	Definitions of data elements, with references to Common Data Elements	N/A
	12	De-identification methods	N/A
	13	How missing data were handled	N/A
Ground Truth	14	Definition of ground truth reference standard, in sufficient detail to allow replication	Yes
	15	Rationale for choosing the reference standard (if alternatives exist)	Yes
	16	Source of ground-truth annotations; qualifications and preparation of annotators	N/A
	17	Annotation tools	N/A
	18	Measurement of inter- and intrarater variability; methods to mitigate variability and/or resolve discrepancies	N/A
Data Partitions	19	Intended sample size and how it was determined	N/A
	20	How data were assigned to partitions; specify proportions	N/A
	21	Level at which partitions are disjoint (e.g., image, study, patient, institution)	N/A
Model	22	Detailed description of model, including inputs, outputs, all intermediate layers and connections	N/A
	23	Software libraries, frameworks, and packages	N/A
	24	Initialization of model parameters (e.g., randomization, transfer learning)	N/A
Training	25	Details of training approach, including data augmentation, hyperparameters, number of models trained	N/A
	26	Method of selecting the final model	N/A
	27	Ensembling techniques, if applicable	N/A
Evaluation	28	Metrics of model performance	N/A
	29	Statistical measures of significance and uncertainty (e.g., confidence intervals)	N/A
	30	Robustness or sensitivity analysis	N/A
	31	Methods for explainability or interpretability (e.g., saliency maps), and how they were validated	N/A
	32	Validation or testing on external data	N/A
RESULTS			
Data	33	Flow of participants or cases, using a diagram to indicate inclusion and exclusion	Yes
	34	Demographic and clinical characteristics of cases in each partition	Yes
Model performance	35	Performance metrics for optimal model(s) on all data partitions	Yes
	36	Estimates of diagnostic accuracy and their precision (such as 95% confidence intervals)	Yes
	37	Failure analysis of incorrectly classified cases	Yes
DISCUSSION			

For peer review only

	**38**	Study limitations, including potential bias, statistical uncertainty, and generalizability	**Yes**
	**39**	Implications for practice, including the intended use and/or clinical role	**Yes**
OTHER INFORMATION			
	**40**	Registration number and name of registry	**N/A**
	**41**	Where the full study protocol can be accessed	**N/A**
	**42**	Sources of funding and other support; role of funders	**Yes**

Supplementary Table 4. CLAIM checklist

For peer review only

Figure 1. Overview of study design. No radiographs or patients were excluded.

Figure 2. **A**: number of patients by age group and sex. **B**: documented patient ethnicities.

Figure 3. Examples of true positive cancerous nodule identification by the software. The circles represent markers placed at the location of the detected abnormality by the AI tool (note that these are not contours of the abnormality). **A**: primary right middle lobe lung cancer. **B**: primary left upper lobe lung cancer. **C**: right upper lobe lung metastasis.

Figure 4. Examples of false negative results. The white circles represent markers placed at the location of the detected abnormality by the AI tool (note that these are not contours of the abnormality). The magenta circles have been added manually to indicate the location of the missed true abnormalities. **A**: missed left lower lobe cancer (magenta circle); the software has also misidentified the left first rib as a false positive abnormality. **B**: missed cancerous left hilar nodule; the software has misidentified the pacemaker as a false positive abnormality.

Figure 5. Examples of false positive abnormalities detected by the software. The circles represent markers placed at the location of the detected abnormality by the AI tool (note that these are not contours of the abnormality). **A**: end of the left first rib. **B**: composite left perihilar shadows. **C**: right nipple shadow. **D**: pacemaker. **E**: old left rib fracture. **F**: right breast implant.

For peer review only

BMJ Open

Evaluating the performance of artificial intelligence software for lung nodule detection on chest radiographs in a retrospective real-world UK population.

Journal:	*BMJ Open*
Manuscript ID	bmjopen-2023-077348.R1
Article Type:	Original research
Date Submitted by the Author:	05-Oct-2023
Complete List of Authors:	Maiter, Ahmed; The University of Sheffield, School of Medicine and Population Health; Sheffield Teaching Hospitals NHS Foundation Trust, Radiology<br>Hocking, Katherine; Sheffield Teaching Hospitals NHS Foundation Trust, Radiology<br>Matthews, Suzanne; Sheffield Teaching Hospitals NHS Foundation Trust<br>Taylor, Jonathan; Sheffield Teaching Hospitals NHS Foundation Trust, 3D Lab<br>Sharkey, Michael; Sheffield Teaching Hospitals NHS Foundation Trust, 3D Lab<br>Metherall, Peter; Sheffield Teaching Hospitals NHS Foundation Trust, 3D Lab<br>Alabed, Samer; The University of Sheffield, School of Medicine and Population Health; Sheffield Teaching Hospitals NHS Foundation Trust, Radiology<br>Dwivedi, Krit; The University of Sheffield, School of Medicine and Population Health; Sheffield Teaching Hospitals NHS Foundation Trust, Radiology<br>Shahin, Yousef; The University of Sheffield, School of Medicine and Population Health; Sheffield Teaching Hospitals NHS Foundation Trust, Radiology<br>Anderson, Elizabeth; Sheffield Teaching Hospitals NHS Foundation Trust, Radiology<br>Holt, Sarah; Sheffield Teaching Hospitals NHS Foundation Trust, Radiology<br>Rowbotham, Charlotte; Sheffield Teaching Hospitals NHS Foundation Trust, Radiology<br>Kamil, Mohamed; Sheffield Teaching Hospitals NHS Foundation Trust, Radiology<br>Hoggard, N; The University of Sheffield, School of Medicine and Population Health; Sheffield Teaching Hospitals NHS Foundation Trust, Radiology<br>Balasubramanian, Saba; Sheffield Teaching Hospitals NHS Foundation Trust<br>Swift, Andrew; The University of Sheffield, School of Medicine and Population Health; Sheffield Teaching Hospitals NHS Foundation Trust, Radiology<br>Johns, Christopher; Sheffield Teaching Hospitals NHS Foundation Trust, Radiology

**Primary Subject Heading**:	Radiology and imaging
Secondary Subject Heading:	Respiratory medicine, Oncology
Keywords:	Diagnostic Imaging, Chest imaging < RADIOLOGY & IMAGING, Diagnostic radiology < RADIOLOGY & IMAGING

I, the Submitting Author has the right to grant and does grant on behalf of all authors of the Work (as defined in the below author licence), an exclusive licence and/or a non-exclusive licence for contributions from authors who are: i) UK Crown employees; ii) where BMJ has agreed a CC-BY licence shall apply, and/or iii) in accordance with the terms applicable for US Federal Government officers or employees acting as part of their official duties; on a worldwide, perpetual, irrevocable, royalty-free basis to BMJ Publishing Group Ltd ("BMJ") its licensees and where the relevant Journal is co-owned by BMJ to the co-owners of the Journal, to publish the Work in this journal and any other BMJ products and to exploit all rights, as set out in our licence.

The Submitting Author accepts and understands that any supply made under these terms is made by BMJ to the Submitting Author unless you are acting as an employee on behalf of your employer or a postgraduate student of an affiliated institution which is paying any applicable article publishing charge ("APC") for Open Access articles. Where the Submitting Author wishes to make the Work available on an Open Access basis (and intends to pay the relevant APC), the terms of reuse of such Open Access shall be governed by a Creative Commons licence – details of these licences and which Creative Commons licence will apply to this Work are set out in our licence referred to above.

Other than as permitted in any relevant BMJ Author's Self Archiving Policies, I confirm this Work has not been accepted for publication elsewhere, is not being considered for publication elsewhere and does not duplicate material already published. I confirm all authors consent to publication of this Work and authorise the granting of this licence.

For peer review only

"Evaluating the performance of artificial intelligence software for lung nodule detection on chest radiographs in a retrospective real-world UK population."

For peer review only

Authors

Dr Ahmed Maiter MB BChir MA[1,2,3], Dr Katherine Hocking MB BChir MA FRCR[1], Dr Suzanne Matthews MBBS MRCP FRCR[1], Dr Jonathan Taylor PhD[2,4], Mr Michael Sharkey MEng MSc[1,4], Dr Peter Metherall PhD[4], Dr Samer Alabed MD PhD FRCR[1,2,3], Dr Krit Dwivedi BMBS BMedSci FRCR[1,2,3] Dr Yousef Shahin MBBS MD FRCR[1,2], Dr Elizabeth Anderson MBBS MRes[1], Sarah Holt MSc[1], Charlotte Rowbotham MSc[1], Dr Mohamed Kamil MBBS FRCR[1], Prof. Nigel Hoggard MD MRCP FRCR[1,2,3], Prof. Saba P Balasubramanian MS PhD FRCS[1], Dr Andy J Swift MBBS PhD FRCR[1,2,3], Dr Christopher S Johns MBBS PhD FRCR[1*]

Affiliations

[1] Sheffield Teaching Hospitals NHS Foundation Trust, Sheffield, UK

[2] Department of Infection, Immunity and Cardiovascular Disease, University of Sheffield, Sheffield, UK

[3] NIHR Sheffield Biomedical Research Centre, Sheffield, UK

[4] 3DLab, Sheffield Teaching Hospitals NHS Foundation Trust, Sheffield, UK

*Corresponding author

Dr Christopher S Johns

Consultant thoracic radiologist

Department of Radiology, Sheffield Teaching Hospitals NHS Foundation Trust, Sheffield, UK

christopher.johns@nhs.net

For peer review only - http://bmjopen.bmj.com/site/about/guidelines.xhtml

For peer review only

For peer review only - http://bmjopen.bmj.com/site/about/guidelines.xhtml

Abstract

Objectives:

Early identification of lung cancer on chest radiographs improves patient outcomes. Artificial intelligence (AI) tools may increase diagnostic accuracy and streamline this pathway. This study evaluated the performance of commercially available AI-based software trained to identify cancerous lung nodules on chest radiographs.

Design:

This retrospective study included primary care chest radiographs acquired in a U.K. centre. The software evaluated each radiograph independently and outputs were compared with two reference standards: 1) the radiologist report and 2) the diagnosis of cancer by multidisciplinary team decision. Failure analysis was performed by interrogating the software marker locations on radiographs.

Participants:

5,722 consecutive chest radiographs were included from 5,592 patients (median age 59 years, 53.8% female, 1.6% prevalence of cancer).

Results:

Compared to radiologist reports for nodule detection, the software demonstrated sensitivity 54.5% [95%CI 44.2-64.4%], specificity 83.2% [82.2-84.1%], positive predictive value 5.5% [4.6-6.6%] and negative predictive value 99.0% [98.8-99.2%]. Compared to cancer diagnosis, the software demonstrated sensitivity 60.1% [50.1-70.1%], specificity 83.3% [82.3-84.2%], positive predictive value 5.6% [4.8-6.6%] and negative predictive value 99.2% [99.0-99.4%]. Normal or variant anatomy was misidentified as an abnormality in 69.9% of the 943 false positive cases.

Conclusions:

The software demonstrated considerable underperformance in this real-world patient cohort. Failure analysis suggested a lack of generalisability in the training and testing datasets as a potential factor. The low positive predictive value carries the risk of over-investigation and limits the translation of the software to clinical practice. Our findings highlight the importance of training and testing software in representative datasets, with broader implications for the implementation of AI tools in imaging.

For peer review only

Keywords

Artificial intelligence; machine learning; computer-aided diagnosis; chest radiograph; lung nodule; lung cancer; diagnostic accuracy

Abbreviations

AI	Artificial intelligence
ALND	Auto Lung Nodule Detection
CI	Confidence interval
CLAIM	Checklist for Artificial Intelligence in Medical Imaging
CT	Computed tomography
FPPI	False positives per image
IQR	Interquartile range
MDT	Multidisciplinary team
NOLCP	National Optimal Lung Cancer Pathway
NPV	Negative predictive value
PACS	Picture Archiving Communications System
PPV	Positive predictive value
RIS	Radiology Information System
UK	United Kingdom

For peer review only

For peer review only - http://bmjopen.bmj.com/site/about/guidelines.xhtml

Strengths and limitations of this study

- The AI software was tested on chest radiographs from a large real-world cohort of patients with an expected prevalence of lung cancer.
- Performance was assessed against two different reference standards: radiologist detection of nodules and diagnosis of lung cancer by multidisciplinary team decision.
- Standard and clinically relevant metrics were used to quantify performance.
- Failure analysis was undertaken to identify factors contributing to incorrect classification of radiographs.
- Limitations include the single-centre retrospective study design, risk of observer bias from manual evaluation of radiograph reports and reliance on documented multidisciplinary team decision as a reference standard.

For peer review only

For peer review only - http://bmjopen.bmj.com/site/about/guidelines.xhtml

Introduction

Early diagnosis of cancer is essential to improve prognosis.[1,2] Lung cancer is the commonest cause of cancer deaths in the United Kingdom (UK), with a 10-year survival rate of only 10%.[3] Early lung cancer may be apparent on chest radiographs as a lung nodule, defined as a focal opacity measuring <30 mm.[4] Although the causes of lung nodules are broad, the detection of a new nodule on a chest radiograph should raise suspicion for cancer and usually necessitates further investigation with CT.[5]

In the UK, the National Optimal Lung Cancer Pathway (NOLCP) was developed to expedite diagnosis and treatment to improve lung cancer survival. The pathway goal is to commence treatment within 49 days of referral for patients with a confirmed diagnosis.[6] According to the NOLCP, patients with chest radiograph abnormalities that are suspicious for lung cancer should undergo diagnostic CT, to be performed and reported within 72 hours. This poses a challenge for healthcare services, particularly in the context of increasing demand for medical imaging and staff shortages.[7] The time taken from radiograph acquisition to reporting can delay the pathway and may be problematic in centres with reporting backlogs. Furthermore, chest radiographs are performed in high volumes for a variety of indications, with only a small proportion raising concern for cancer.

The application of artificial intelligence (AI) methods to medical imaging is expanding rapidly and promises a number of benefits.[8-10] Automated evaluation of images could help to triage studies for earlier reporting by radiologists, focusing resource allocation to cases where timely reporting is likely to have the largest clinical benefit. Recent years have seen an increase in the number of studies presenting AI approaches to the evaluation of chest radiographs, either alone or in combination with radiologists. These have included tools to assist with the identification of acute abnormalities such as pneumonia or pneumothorax,[11-14] tuberculosis screening,[15-17] and identification of lung cancer.[18-20] Studies have also evaluated how AI-based categorisation of chest radiographs could help to streamline clinical workflows.[21,22] As the field advances and AI tools become commercially available, it is imperative that their performance in real-world populations and clinical settings is evaluated and understood.

The Auto Lung Nodule Detection software (ALND; Samsung Electronics, Suwon, South Korea) was developed to aid identification of lung nodules on digital chest radiographs. The software was Class IIa medical devices directive UKCA and CE-marked, FDA-approved, and commercially available at the time of our study. The design, training and validation of the software has been reported previously.[23,24] The software is based on a deep

For peer review only - http://bmjopen.bmj.com/site/about/guidelines.xhtml

convolutional neural network algorithm, trained using an enriched dataset of 17,210 radiographs (79·7% normal and 20·3% with cancerous lung nodules) from a single South Korean centre. The software was subsequently tested on 800 radiographs (25·0% normal and 75·0% with cancerous lung nodules) from four external datasets (from South Korea, Germany and the United States). The performance when used alone varied between the four enriched datasets, with a sensitivity range of 51·1% to 79·1% and false positives per image (FPPI) rate of 0·10 to 0·30. However, the software has not been tested in an unselected real-world cohort of patients attending for routine chest radiographs.

This study aimed to evaluate the performance of ALND at detecting lung nodules on chest radiographs seen in routine practice in a tertiary UK centre. Radiologist reports and lung cancer multidisciplinary team (MDT) decisions were used as the reference standards for lung nodule and lung cancer diagnosis.

For peer review only - http://bmjopen.bmj.com/site/about/guidelines.xhtml

Materials and methods

This retrospective single-centre study was undertaken at Sheffield Teaching Hospitals NHS Foundation Trust, Sheffield, U.K. Study approval was obtained from the local clinical research office, a local ethics committee (Sheffield 3DLab; reference 17/YH/0142), and information governance team. Requirement for direct patient consent was waived by the clinical research office and ethics committee. The study flow is indicated in **Figure 1**. The study is presented in accordance with the Checklist for Artificial Intelligence in Medical Imaging (CLAIM; Supplementary Materials).[25] Data analysis was performed using Prism (GraphPad, San Diego, CA, USA) and R Studio using the Tidyverse and EpiR packages.

1. Case selection

Chest radiographs were identified retrospectively from the local radiology information system. Radiographs were eligible for inclusion if they met all of the following criteria: 1) requested by the patient's general practitioner, 2) performed on adult patients 3) between July 1 2020 and February 26 2021, and acquired 3) in the posterior-anterior projection, 4) on one of three GC85A digital radiography systems (Samsung Electronics, Suwon, South Korea) running the software tool (ALND v1·0, Samsung Electronics, Suwon, South Korea) 5) at the same tertiary centre (Royal Hallamshire Hospital, Sheffield, UK).

2. Evaluation of clinical reports

For each included radiograph, the clinical report was assessed retrospectively for an opinion on the presence of a suspicious nodule or other features of cancer. This was performed manually given the broad variation in reporting styles and language. For example, reports could identify a suspicious nodule explicitly or implicitly (e.g. 'two-week wait referral is advised' or "urgent CT is recommended").

3. Software results

Included radiographs were assessed by the software on the digital radiography system at the time of acquisition. For each radiograph, the software outputs included the original DICOM-formatted image, a binary assessment for the presence of a suspicious lung nodule (i.e. 'yes' or 'no'), the number of detected abnormalities and a copy of the image with a marker on each detected abnormality (*not* a contour of the abnormality, but only an indication

For peer review only - http://bmjopen.bmj.com/site/about/guidelines.xhtml

of location). The outputs were sent to a research-only Picture Archiving Communications System (PACS) and were not accessible for clinical use.

4. Accuracy of nodule detection

For each case, the software output was compared with the corresponding clinical report, which was used as the reference standard for the presence of a suspicious nodule. Sensitivity, specificity, positive predictive value (PPV), negative predictive value (NPV), overall accuracy and FPPI were calculated from contingency tables for all cases. FPPI was calculated as the number of false positive results divided by the number of radiographs.

5. Accuracy of cancer diagnosis

Records from the local lung cancer multidisciplinary team (MDT) meetings were reviewed from the date of the first included radiograph to six months after the last included radiograph. Cases with a diagnosis of cancer by lung MDT decision were identified. Diagnosis of lung cancer by MDT opinion was used as the reference standard, against which the software output and clinical report were compared for each case. Sensitivity, specificity, PPV, NPV, overall accuracy and FPPI were calculated from contingency tables. In those cases where a cancer was identified by the software, the output was interrogated to ensure that the identified abnormality was in the correct location; if the marker had been placed incorrectly by the software this was counted as a negative result. For each case with a diagnosis of cancer, the radiograph was re-reviewed by one consultant thoracic radiologist (CSJ, 11 years of experience) to determine if the abnormality was visible; subgroup analysis was performed excluding cases in which the cancerous abnormality was not visible on the radiograph.

6. Failure analysis of incorrectly classified cases

Failure analysis was performed for false positive and false negative outputs by the software in reference to the presence or absence of MDT-confirmed cancer. For each of these cases, the software outputs were manually compared with the radiograph, clinical report and MDT opinion.

7. Patient and public involvement

There was no patient or public involvement in this study.

For peer review only

For peer review only - http://bmjopen.bmj.com/site/about/guidelines.xhtml

Results

1. Included radiographs and patients

A total of 5,722 chest radiographs were included from 1 July 2020 to 26 February 2021. These were acquired for 5,592 different patients, with 2·3% of these having had either two or three radiographs. The included patients had a median age of 59 years (IQR 46-72 years), with 53·8% female (**Figure 2A**). 79·0% of patients were white or white British. Ethnicity information was unavailable for 9·1% of cases (**Figure 2B**). No radiographs or patients were excluded.

2. Clinical reports

All radiographs had been reported independently by FRCR-qualified radiologists, with 91·0% reported by 7 different consultant thoracic radiologists. 58·5% of reports mentioned comparison with previous imaging studies. Potentially cancerous abnormalities were identified in 3·0% of reports; these included nodules (59·1%), masses (25·7%) and other abnormalities such as lung volume loss or pleural thickening (21·6%).

3. Accuracy of nodule detection

All 5,722 chest radiographs were evaluated by the software, with none failing evaluation. The software identified a total of 1,120 potentially cancerous nodules on 17·5% of radiographs. When compared against identification of suspicious nodules in the clinical report (i.e. excluding masses or other abnormalities for which the software had not been trained), the software yielded a sensitivity of 54·5%, specificity of 83·2%, PPV of 5·5%, NPV of 99·0% and overall accuracy of 82·7%, with a FPPI of 0·18 (**Table 1** and **Supplementary Table 1A**).

For peer review only

Test	Suspicious nodule identified by software	Suspicious nodule identified by software	Any suspicious abnormality on clinical report**
**Reference standard**	Suspicious nodule on clinical report*	Cancer diagnosis by MDT decision	
**Sensitivity**	54·5% [44·2 – 64·4]	60·9% [50·1 – 70·9]	66·3% [55·7 – 75·8]
**Specificity**	83·2% [82·2 – 84·1]	83·3% [82·3 – 84·2]	98·0% [97·7 – 98·4]
**PPV**	5·5% [4·6 – 6·6]	5·6% [4·8 – 6·6]	35·7% [30·5 – 41·2]
**NPV**	99·0% [98·8 – 99·2]	99·2% [99·0 – 99·4]	99·4% [99·3 – 99·6]
**Accuracy**	82·7% [81·7 – 83·6]	82·9% [81·9 – 83·9]	97·5% [97·1 – 97·9]
**FPPI**	0·18	0·18	0·02

Table 1. Performance of the software and clinical reports against reference standards, with 95% confidence intervals. PPV = positive predictive value, NPV = negative predictive value, FPPI = false positives per image. *Other suspicious abnormalities (including masses and secondary features of cancer, such as lung volume loss) were excluded. **Includes nodules, masses, and secondary features of malignancy (such as unilateral pleural effusions or lobar collapse).

4. Accuracy of cancer diagnosis

92 patients (1·6%) were found to have a diagnosis of cancer by MDT opinion. When the suspicious nodules identified by the software were compared against MDT diagnosis, the software yielded a sensitivity of 60·9%, specificity of 83·3%, PPV of 5·6%, NPV of 99·2% and accuracy of 82·9%, with a FPPI of 0·18 (**Table 1** and **Supplementary Table 1B**). Examples of correct identification of cancer by the software are shown in Figure 3. When all suspicious abnormalities identified in the clinical reports (i.e. including nodules, masses and other abnormalities) were compared against MDT diagnosis, the clinical reports yielded a sensitivity of 66·3%, specificity of 98·0%, PPV of 35·7%, NPV of 99·4% and accuracy of 97·5%, with a FFPI of 0·02 (**Table 1** and

For peer review only - http://bmjopen.bmj.com/site/about/guidelines.xhtml

Supplementary Table 1C). Hypothetically, if the detection of an abnormality automatically resulted in patients undergoing CT, the software would have resulted in 999 scans with 60·9% of cancer cases detected (**Supplementary Table 1B**). By comparison, the clinical reports would have resulted in 171 scans with 66·3% of cancer cases detected. (**Supplementary Table 1C**).

Of the 92 cases of cancer, the abnormality was deemed to be visible on the radiograph in 75 cases. Using these cases as the reference standard, the software yielded a sensitivity of 68·0%, specificity of 83·3%, PPV of 5·1%, NPV of 99·5% and accuracy of 83·0% (**Supplementary Table 1D** and **Supplementary Table 2**). The clinical reports yielded a sensitivity of 80·0%, specificity of 98·0%, PPV of 35·3%, NPV of 99·7% and accuracy of 97·8%. (**Supplementary Table 1E and Supplementary Table 2**). Performance of the clinical reports was similar to expected.[26]

5. Failure analysis of ALND results

The software yielded 34 false negatives compared to MDT opinion (36·9% of cases with cancer). In 22 of these cases, the abnormalities were found to be visible on the radiograph and included masses (63·5 %, defined as >30 mm), lung collapse secondary to obstructing cancer (18·2 %), solitary or multiple cancerous nodules (13·6%), malignant pleural effusion (9·1%) and persistent consolidation (9·1%) (**Figure 4**).

The software yielded 943 false positive results in comparison with MDT opinion, representing 16·8% of all cases without cancer. Only 7·8% of these cases had been reported as abnormal in the formal clinical report and were subsequently found to be due to a benign cause. Contrastingly, the formal clinical report yielded 110 false positive results compared to MDT opinion, representing 2·0% of cases without cancer. Causes of failure by the software included normal anatomy (69·9%), non-cancerous pathology (31·2%) and technical factors (4·8%) (**Figure 5** and **Supplementary Table 3**).

For peer review only - http://bmjopen.bmj.com/site/about/guidelines.xhtml

Discussion

The evaluation of AI tools in real-world settings is essential for their translation to clinical practice. In this retrospective study, we evaluated the performance of an AI-based software tool trained to identify suspicious lung nodules on chest radiographs. The software demonstrated underperformance on consecutive patients referred from primary care. While sensitivity and FFPI values were similar to those reported previously for enriched datasets, we have demonstrated a considerably low PPV when tested in a real-world dataset. The results carry important implications for the training, evaluation, and clinical implementation of AI tools in medical imaging.

A large number of consecutive cases referred from primary care were included using broad inclusion criteria. We believe that this unenriched dataset is more representative of the population encountered in routine clinical practice. The software demonstrated low PPV values, which severely limit the potential utility of the software and make it unsuitable for our population. Compared to radiologist reports, the software underperformed in all measures of diagnostic performance. Our results raise important questions about the intended use of the software and how it could fit into a clinical pathway. The low PPV demonstrated here suggests that use of the software for its intended purpose would result in over-investigation of patients without an improvement in sensitivity. In comparison with the clinical reports, if all cases where the software had detected an abnormality suspicious for cancer had undergone CT, this would have resulted in 5.8 times the number of scans performed and fewer cases of cancer detected. Such over-investigation is harmful: unnecessary diagnostic tests carry physical risks such as increased doses of ionising radiation, may contribute to patient anxiety and distress, and can increase the demands on healthcare systems. Our results highlight the difference between AI *model* performance and *clinical* performance. The software yielded similar sensitivity and FPPI results to those during its previous validation and testing in enriched datasets,[23,24] but showed very low PPV when applied to a real-world population with normal disease prevalence, representing underperformance for its intended clinical purpose.

Understanding *why* underperformance has occurred is essential for further development of AI tools. As part of the output for each radiograph, the software labelled detected abnormalities. This is an important design feature, enabling failure analysis, allowing proper evaluation of performance, and aiding decision-making if used in clinical settings. The software frequently misidentified normal, variant, or age-related anatomy as abnormalities. This included false positives that radiologists readily dismiss, such as the ends of the first ribs. What factors could have contributed to this? One potential explanation is the use of insufficiently representative and generalisable datasets for software training and testing. The training and testing datasets were enriched and included a

For peer review only

prevalence of cancer far higher than that seen in real world clinical populations, which may have skewed performance towards overcalling abnormalities.[23] In contrast, our dataset had a more realistic prevalence of 1·6%. Differences in population demographics may have also contributed to underperformance. The software was trained on a South Korean population.[23] Gichoya *et al.* recently demonstrated that AI tools are capable of determining a patient's race from radiographs, highlighting that there can be an inherent difference in normal imaging appearances between populations.[27,28] An AI model trained on data from one population may not generalise well to others that show significant demographic differences. The interaction of AI with patient factors such as sex, race, and ethnicity is an important practical and ethical issue for the field moving forwards. Alternatively, insufficient variation and volume in the 'normal' radiographs used in training may have affected performance. The spectrum of normality is broad and can encompass variations in anatomy and technical factors relating to radiograph acquisition. How 'normal' radiographs were defined in the training and testing datasets was not reported clearly. For example, did these include anatomical variants, age-related changes, radiographs that were rotated, or artefacts from external clothing - all of which are seen commonly in routine practice? And if so, were these included in a sufficient and representative number? Furthermore, the training and testing datasets did not include non-cancerous pathology. For example, smoking and industrial exposures are risk factors for lung cancer and cause benign abnormalities that are visible on chest radiographs, such as emphysema or pleural plaques. It is important for an AI tool to be able to identify cancer in a population of patients with co-existing pathologies that reflect an increased cancer risk. Masses and secondary features of cancer (such as pleural effusion or mediastinal lymphadenopathy) were also absent from the training and testing datasets. Finally, the software was trained to assess radiographs in isolation, with no comparison to previous imaging or clinical context, in stark contrast to how radiologists interpret imaging. In short, training and testing datasets need to be representative of the intended population to ensure generalisability of AI tools, and models should ideally integrate information from previous imaging studies, patient demographics and clinical history. While existing studies have often used enriched data sets to develop and test models, promising models should be tested in representative real-world datasets and environments prior to their routine clinical use.

Our study has limitations. The retrospective design enabled a large study population but did not afford the same degree of robustness or control over confounding variables as a prospective study. Using data from routine clinical care risks incomplete information, such as a lack of documented ethnicity in some patients. There is also inherent difficulty in defining the reference standard for a suspicious nodule. Other studies have used CT or biopsy results, but such approaches are less practical when evaluating a large real-world clinical cohort and introduce their own

For peer review only - http://bmjopen.bmj.com/site/about/guidelines.xhtml

selection bias: some inflammatory nodules will be transient and not warrant further investigation, some patients may not undergo CT or biopsy, or some results may be indeterminate. We used cancers diagnosed by lung MDT decision as a reference standard, but some cases of cancer may have been missed if they had not been referred to or reviewed by the MDT, or if they represented metastatic disease to the lung from another known primary cancer. Finally, assessment of the clinical reports and failure analysis were performed by radiologists, with the risk of interobserver variability or observer bias. Regardless, we believe that the study limitations are unlikely to explain the considerable differences in performance compared to the software's prior validation and testing. Looking ahead, it is important that software tools showing promising performance in representative datasets such as ours are then more formally evaluated prospectively, such as through randomised controlled trials.

The excitement surrounding AI is understandable given the promise of automation in an era of growing demands for medical imaging. The Royal College of Radiologists reports a shortfall of 29% of the UK radiologist workforce, raising concerns about reporting backlogs and the potential for delayed diagnoses.[7] Chest radiographs remain a mainstay of imaging in radiology departments, with a median of just under 650,000 performed per month in England in 2021-2022.[29] The ability to streamline reporting would assist in dealing with this high burden, addressing backlogs and minimising the impacts of service pressures. Early identification of patients with possible lung cancer on chest radiographs would facilitate more efficient workflows, reducing the time to diagnosis and improving patient outcomes.[1,2] However, this study highlights fundamental questions about the application of AI in medical imaging. Firstly, what constitutes sufficient training and testing? We have demonstrated that performance in narrow and enriched datasets during software development is not necessarily generalisable to real-world populations. This issue is likely to become more significant as the field advances and model performances improve.[21] However, when the pre-test probability of the disease is low, large population samples will be required to demonstrate significant differences in performance. There remain significant ethical concerns about the systematic propagation of human bias in AI tools that may disadvantage minority populations and datasets need to be selected carefully and presented in a transparent manner.[27,28,30,31] Secondly, what are the appropriate benchmarks for performance? Proper definition of reference standards can be challenging, particularly in clinical settings. Radiologist opinion is a common reference standard for medical imaging AI tools and is an intuitive benchmark when considering the utility of a tool, but is subject to its own internal error rate and both intra-observer and inter-observer variability. In this study, we have not only compared the software performance against radiologist reports, but also MDT diagnosis of lung cancer, which accounted for clinical, imaging, and histopathological factors. The use of MDT diagnosis as a reference standard reflects real-life clinical

For peer review only

practice and is an appropriate benchmark when considering the clinical utility of AI tools for cancer detection. Thirdly, how should performance be presented and interpreted? High-quality reporting of model design, training, validation, and testing is crucial for understanding the performance and generalisability of AI tools.[32] Transparency of AI tool development and performance is essential for the gaining the trust of stakeholders, including the public, and is therefore important for translation of tools into the clinical sphere. The way AI tools are presented should be consistent, enabling direct comparisons of performance, and accessible to all stakeholders, allowing purpose and performance to be understood by those without extensive experience in the field of AI. Poor performance should also be presented, analysed, and published in a fair manner to minimise bias within the field. Lastly, where do we see such AI tools fitting into clinical pathways? We found a low PPV, which combined with a low prevalence of disease in the studied population, suggest that implementation of the software would result in over-investigation and potentially *contributing* to the burden on healthcare systems. Conversely, the NPV of the software exceeded 99%, and there could be potential utility in using the software to streamline reporting by removing studies that do not have cancer. Other studies have presented the use of AI approaches to categorise chest radiographs for the purpose of report prioritisation, which is of particular appeal given the large volumes requiring reporting.[21,22] However, in this case the software would also exclude non-cancerous abnormalities that could still be significant (such as infection) and would offer no improvement over a radiologist's NPV, and therefore may not add much clinical value. Alternatively, AI tools may be of benefit when used to augment reporting by radiologists or to provide a second reading.[13,33,34] In order to streamline workflows and pathways, performance thresholds need to be chosen carefully and determined prior to software development.

Conclusion

Evaluation of AI-based software tools in representative patient cohorts is crucial for their translation to routine clinical practice. We have demonstrated underperformance of the software using a large retrospective and relatively unselected clinical cohort, despite apparent similar diagnostic accuracy in comparison to the initial training and testing. Understanding the external validity of software performance is an important consideration as the field continues to advance.

For peer review only - http://bmjopen.bmj.com/site/about/guidelines.xhtml

Figure legends

Figure 1. Overview of study design. No radiographs or patients were excluded.

Figure 2. **A**: number of patients by age group and sex. **B**: documented patient ethnicities.

Figure 3. Examples of true positive cancerous nodule identification by the software. The circles represent markers placed at the location of the detected abnormality by the AI tool (note that these are not contours of the abnormality). **A**: primary right middle lobe lung cancer. **B**: primary left upper lobe lung cancer. **C**: right upper lobe lung metastasis.

Figure 4. Examples of false negative results. The white circles represent markers placed at the location of the detected abnormality by the AI tool (note that these are not contours of the abnormality). The magenta circles have been added manually to indicate the location of the missed true abnormalities. **A**: missed left lower lobe cancer (magenta circle); the software has also misidentified the left first rib as a false positive abnormality. **B**: missed cancerous left hilar nodule; the software has misidentified the pacemaker as a false positive abnormality.

Figure 5. Examples of false positive abnormalities detected by the software. The circles represent markers placed at the location of the detected abnormality by the AI tool (note that these are not contours of the abnormality). **A**: end of the left first rib. **B**: composite left perihilar shadows. **C**: right nipple shadow. **D**: pacemaker. **E**: old left rib fracture. **F**: right breast implant.

For peer review only

For peer review only - http://bmjopen.bmj.com/site/about/guidelines.xhtml

Author contributions

CSJ, SM, PM and **JT** conceptualised and designed the study. **PM** developed software for data curation and analysis. **CR**, **SJH** and **CSJ** undertook data curation. **KH**, **SJH, YS** and **EA** extracted information from the dataset. **CSJ**, **SM** and **KH** performed image failure analysis. **CSJ** and **AM** performed formal data analysis for all data. **AM** and **CSJ** wrote the original manuscript, which was edited and reviewed by **KH**, **AJS**, **SA**, **NH**, **SPB, CR** and **MAK**. **AM** and **CSJ** produced the final manuscript. **All authors** have read and approved the final version of the manuscript. **AM**, **CSJ** and **KH** had direct access to and verified the data presented in the manuscript. **AM** and **CSJ** revised the manuscript following peer review.

Conflicts of interest

The authors have no conflicts of interest to disclose.

Funding

Samsung Electronics (Suwon, South Korea) provided free access to the Auto Lung Nodule Detection (ALND) software for the Sheffield Teaching Hospitals for the purposes of this study. AJS is supported by a Wellcome Trust Fellowship Grant (205188/Z/16/Z). KD is supported by a Wellcome Trust Fellowship Grant (222930/Z/21/Z and 4ward North 203914/Z/16/). This research was carried out in part at the National Institute for Health and Care Research (NIHR) Sheffield Biomedical Research Centre (BRC). The funders did not have any role in the design and conduct of the study; in the collection, analysis, and interpretation of the data; or in the preparation, review, and approval of the paper. The views expressed are those of the author(s) and not necessarily those of the funders.
For the purpose of Open Access, the author has applied a CC BY public copyright licence to any Author Accepted Manuscript version arising from this submission.

Data sharing

Anonymised participant data can be provided on reasonable request pending local information governance approval, which may require a signed data access agreement.

For peer review only - http://bmjopen.bmj.com/site/about/guidelines.xhtml

References

1 Yang C-FJ, Wang H, Kumar A, *et al.* Impact of Timing of Lobectomy on Survival for Clinical Stage IA Lung Squamous Cell Carcinoma. *Chest* 2017; **152**: 1239–50.

2 Navani N, Nankivell M, Lawrence DR, *et al.* Lung cancer diagnosis and staging with endobronchial ultrasound-guided transbronchial needle aspiration compared with conventional approaches: an open-label, pragmatic, randomised controlled trial. *Lancet Respir Med* 2015; **3**: 282–9.

3 Survival | Lung cancer | Cancer Research UK. https://www.cancerresearchuk.org/about-cancer/lung-cancer/survival (accessed Dec 10, 2022).

4 Quadrelli S, Lyons G, Colt H, Chimondeguy D, Buero A. Clinical Characteristics and Prognosis of Incidentally Detected Lung Cancers. *Int J Surg Oncol* 2015; **2015**: e287604.

5 National Institute for Health and Care Excellence (NICE). Lung cancer: diagnosis and management. https://www.nice.org.uk/guidance/ng122 (accessed Dec 10, 2022).

6 NHS England. Rapid cancer diagnostic and assessment pathways. https://www.england.nhs.uk/publication/rapid-cancer-diagnostic-and-assessment-pathways/ (accessed Dec 10, 2022).

7 The Royal College of Radiologists. Clinical radiology census report 2021. 2022. https://www.rcr.ac.uk/clinical-radiology/rcr-clinical-radiology-census-report-2021 (accessed Jan 5, 2023).

8 Hosny A, Parmar C, Quackenbush J, Schwartz LH, Aerts HJWL. Artificial intelligence in radiology. *Nat Rev Cancer* 2018; **18**: 500–10.

9 Killock D. AI outperforms radiologists in mammographic screening. *Nat Rev Clin Oncol* 2020; **17**: 134–134.

10 Neri E, de Souza N, Brady A, *et al.* What the radiologist should know about artificial intelligence – an ESR white paper. *Insights Imaging* 2019; **10**: 44.

11 Hwang EJ, Park S, Jin K-N, *et al.* Development and Validation of a Deep Learning–Based Automated Detection Algorithm for Major Thoracic Diseases on Chest Radiographs. *JAMA Netw Open* 2019; **2**: e191095.

12 Singh R, Kalra MK, Nitiwarangkul C, *et al.* Deep learning in chest radiography: Detection of findings and presence of change. *PloS One* 2018; **13**: e0204155.

13 Choi SY, Park S, Kim M, Park J, Choi YR, Jin KN. Evaluation of a deep learning-based computer-aided detection algorithm on chest radiographs: Case-control study. *Medicine (Baltimore)* 2021; **100**: e25663.

14 Nam JG, Kim M, Park J, *et al.* Development and validation of a deep learning algorithm detecting 10 common abnormalities on chest radiographs. *Eur Respir J* 2021; **57**. DOI:10.1183/13993003.03061-2020.

15 Codlin AJ, Dao TP, Vo LNQ, *et al.* Independent evaluation of 12 artificial intelligence solutions for the detection of tuberculosis. *Sci Rep* 2021; **11**: 23895.

16 Lee JH, Park S, Hwang EJ, *et al.* Deep learning-based automated detection algorithm for active pulmonary tuberculosis on chest radiographs: diagnostic performance in systematic screening of asymptomatic individuals. *Eur Radiol* 2021; **31**: 1069–80.

17 Nash M, Kadavigere R, Andrade J, *et al.* Deep learning, computer-aided radiography reading for tuberculosis: a diagnostic accuracy study from a tertiary hospital in India. *Sci Rep* 2020; **10**: 210.

18 Yoo H, Kim KH, Singh R, Digumarthy SR, Kalra MK. Validation of a Deep Learning Algorithm for the Detection of Malignant Pulmonary Nodules in Chest Radiographs. *JAMA Netw Open* 2020; **3**: e2017135.

For peer review only - http://bmjopen.bmj.com/site/about/guidelines.xhtml

19 Koo YH, Shin KE, Park JS, Lee JW, Byun S, Lee H. Extravalidation and reproducibility results of a commercial deep learning-based automatic detection algorithm for pulmonary nodules on chest radiographs at tertiary hospital. *J Med Imaging Radiat Oncol* 2021; **65**: 15–22.

20 Nam JG, Park S, Hwang EJ, *et al.* Development and Validation of Deep Learning–based Automatic Detection Algorithm for Malignant Pulmonary Nodules on Chest Radiographs. *Radiology* 2019; **290**: 218–28.

21 Dyer T, Dillard L, Harrison M, *et al.* Diagnosis of normal chest radiographs using an autonomous deep-learning algorithm. *Clin Radiol* 2021; **76**: 473.e9-473.e15.

22 Annarumma M, Withey SJ, Bakewell RJ, Pesce E, Goh V, Montana G. Automated Triaging of Adult Chest Radiographs with Deep Artificial Neural Networks. *Radiology* 2019; **291**: 196–202.

23 Sim Y, Chung MJ, Kotter E, *et al.* Deep Convolutional Neural Network–based Software Improves Radiologist Detection of Malignant Lung Nodules on Chest Radiographs. *Radiology* 2020; **294**: 199–209.

24 Jacobson FL. Medical Image Perception Research in the Emerging Age of Artificial Intelligence. *Radiology* 2020; **294**: 210–1.

25 Mongan J, Moy L, Kahn CE. Checklist for Artificial Intelligence in Medical Imaging (CLAIM): A Guide for Authors and Reviewers. *Radiol Artif Intell* 2020; **2**: e200029.

26 Aberle DR, DeMello S, Berg CD, *et al.* Results of the Two Incidence Screenings in the National Lung Screening Trial. *N Engl J Med* 2013; **369**: 920–31.

27 Gichoya JW, Banerjee I, Bhimireddy AR, *et al.* AI recognition of patient race in medical imaging: a modelling study. *Lancet Digit Health* 2022; **4**: e406–14.

28 Wiens J, Creary M, Sjoding MW. AI models in health care are not colour blind and we should not be either. *Lancet Digit Health* 2022; **4**: e399–400.

29 NHS England. Diagnostic Imaging Dataset 2021-22 Data. https://www.england.nhs.uk/statistics/statistical-work-areas/diagnostic-imaging-dataset/diagnostic-imaging-dataset-2021-22-data/ (accessed Jan 5, 2023).

30 Seyyed-Kalantari L, Zhang H, McDermott MBA, Chen IY, Ghassemi M. Underdiagnosis bias of artificial intelligence algorithms applied to chest radiographs in under-served patient populations. *Nat Med* 2021; **27**: 2176–82.

31 Char DS, Shah NH, Magnus D. Implementing Machine Learning in Health Care — Addressing Ethical Challenges. *N Engl J Med* 2018; **378**: 981–3.

32 Alabed S, Maiter A, Salehi M, *et al.* Quality of reporting in AI cardiac MRI segmentation studies – A systematic review and recommendations for future studies. *Front Cardiovasc Med* 2022; **9**. https://www.frontiersin.org/articles/10.3389/fcvm.2022.956811 (accessed Jan 6, 2023).

33 Tam MDBS, Dyer T, Dissez G, *et al.* Augmenting lung cancer diagnosis on chest radiographs: positioning artificial intelligence to improve radiologist performance. *Clin Radiol* 2021; **76**: 607–14.

34 McKinney SM, Sieniek M, Godbole V, *et al.* International evaluation of an AI system for breast cancer screening. *Nature* 2020; **577**: 89–94.

For peer review only

For peer review only - http://bmjopen.bmj.com/site/about/guidelines.xhtml

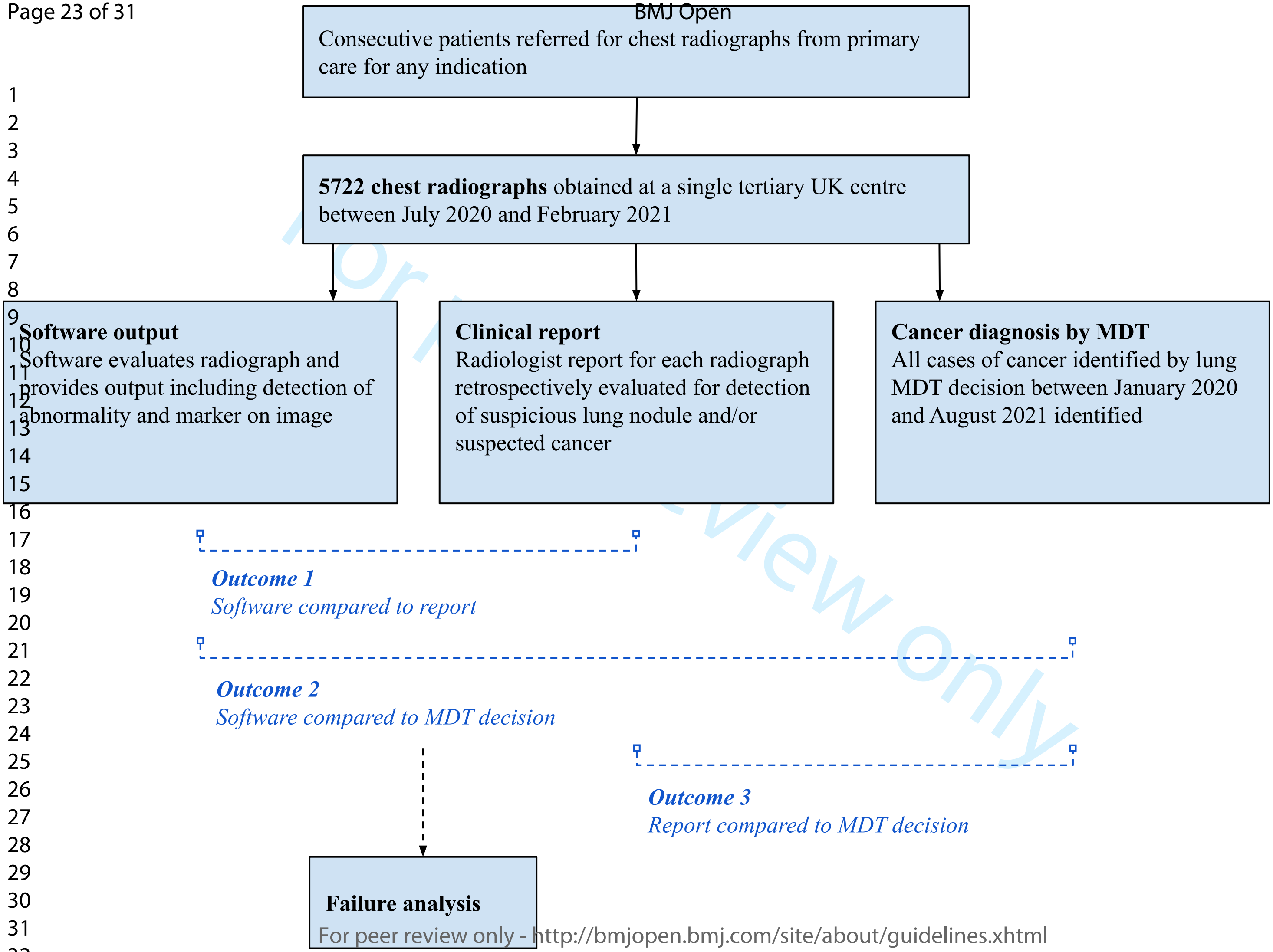

For peer review only

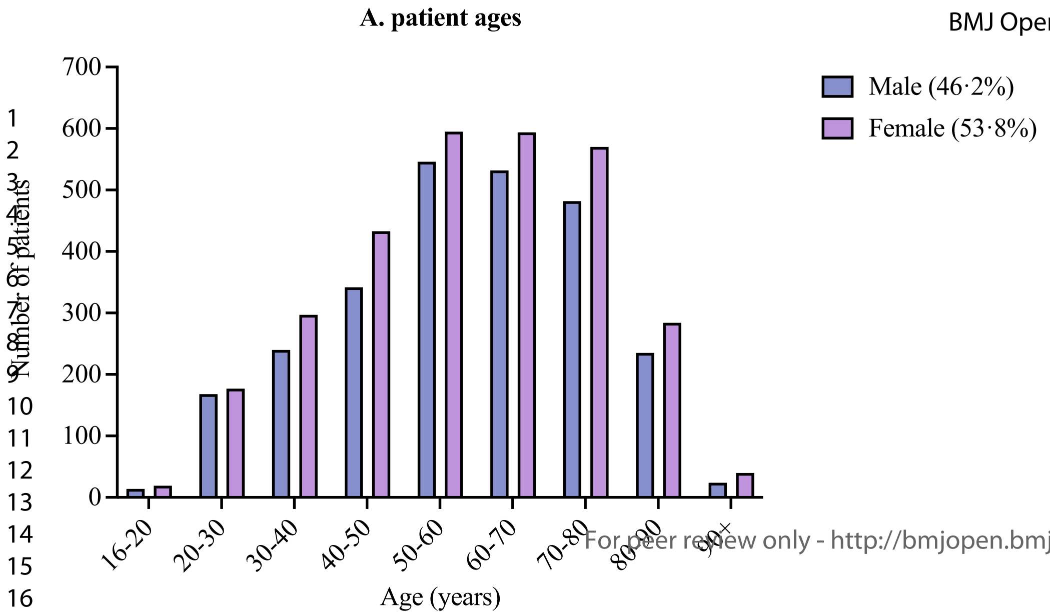

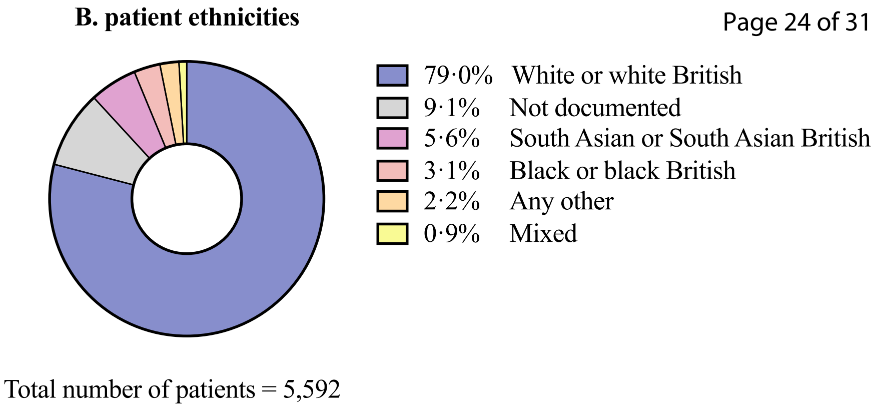

For peer review only - http://bmjopen.bmj.com/site/about/guidelines.xhtml

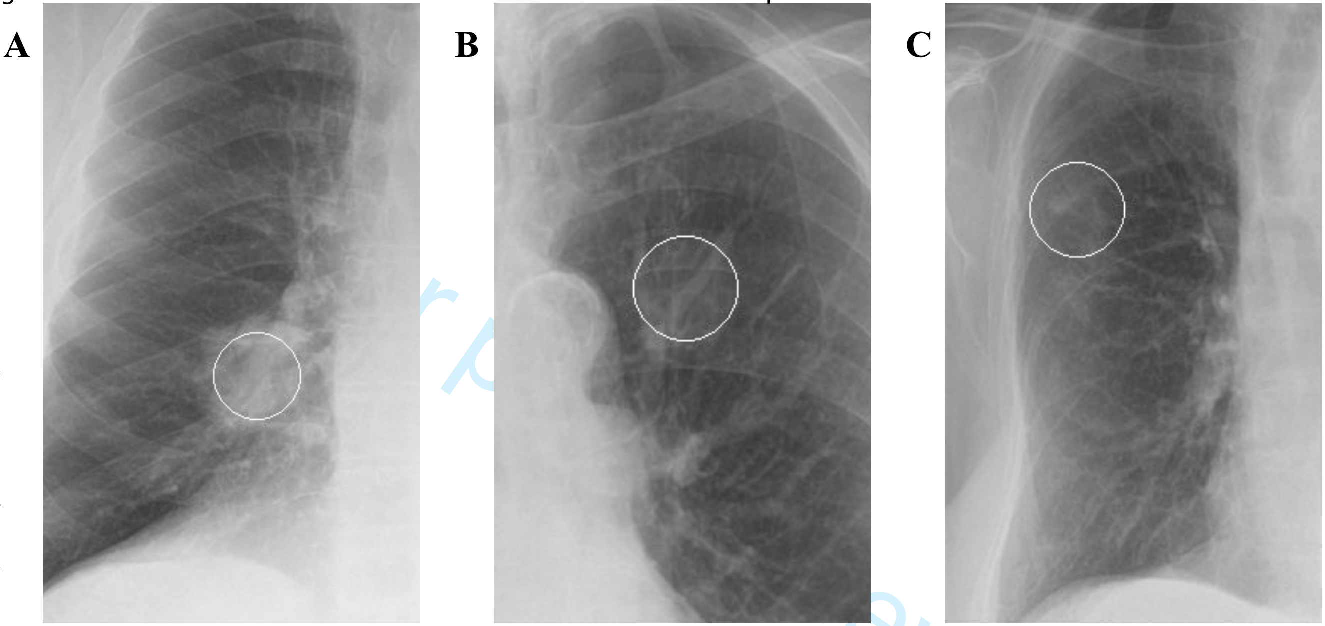
A
B
C

A

B

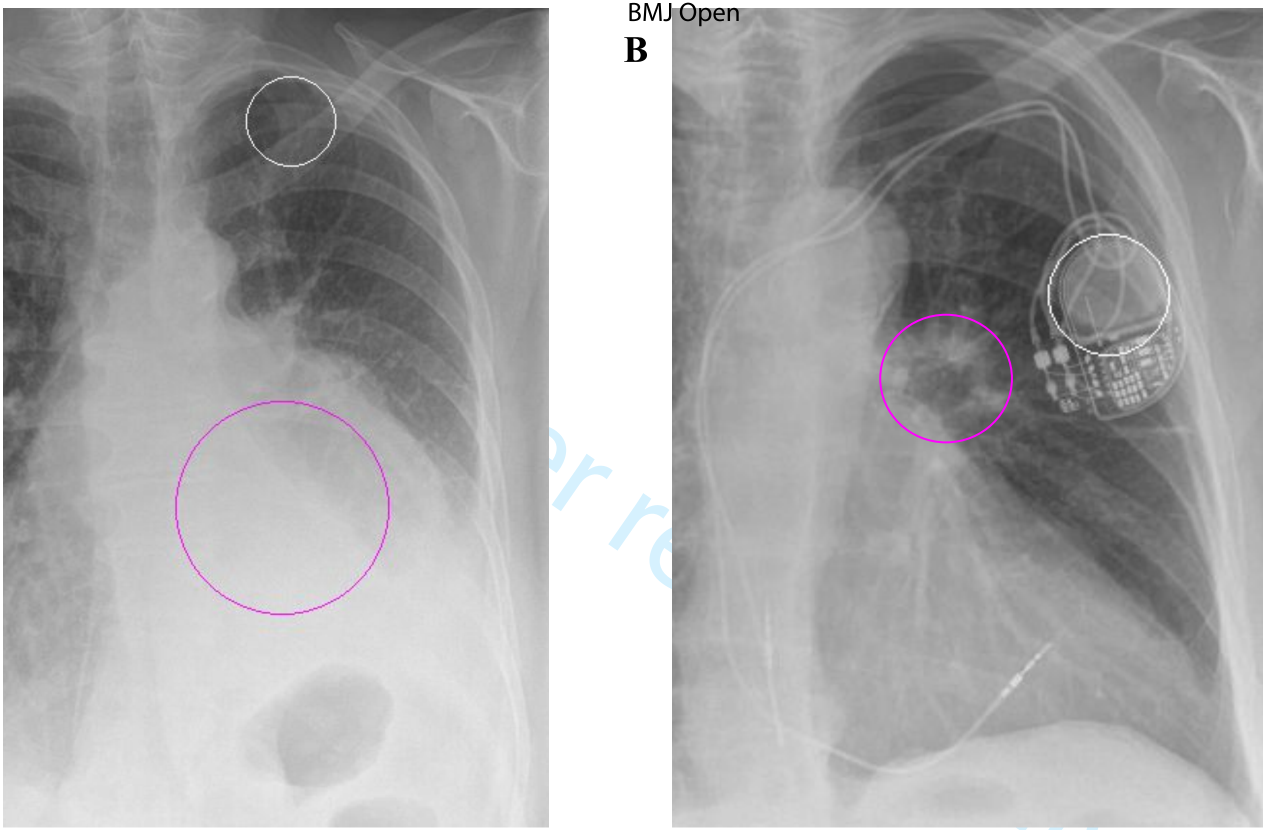

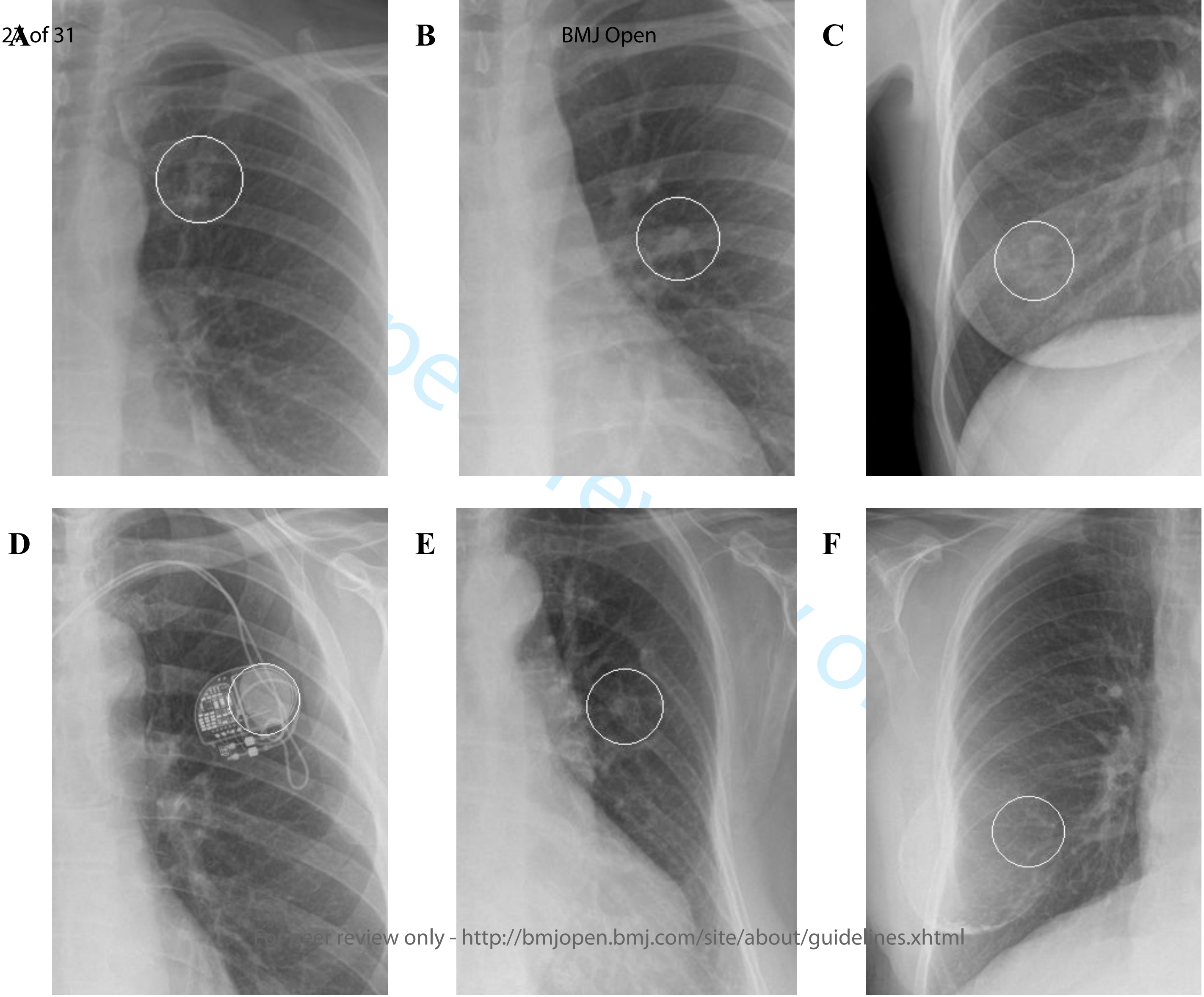
A
B
C
D
E
F

A. accuracy of nodule detection by software		Clinical report REFERENCE STANDARD Positive	Clinical report REFERENCE STANDARD Negative
**ALND software** TEST	**Positive**	55 TRUE POSITIVE	946 FALSE POSITIVE
	**Negative**	45 FALSE NEGATIVE	4676 TRUE NEGATIVE

B. accuracy of cancer detection by software		MDT-reported cancer REFERENCE STANDARD Positive	MDT-reported cancer REFERENCE STANDARD Negative
**ALND software** TEST	**Positive**	56 TRUE POSITIVE	943 FALSE POSITIVE
	**Negative**	36 FALSE NEGATIVE	4687 TRUE NEGATIVE

C. accuracy of cancer detection by clinical reports		MDT-reported cancer REFERENCE STANDARD Positive	MDT-reported cancer REFERENCE STANDARD Negative
**Clinical report** TEST	**Positive**	61 TRUE POSITIVE	110 FALSE POSITIVE
	**Negative**	31 FALSE NEGATIVE	5520 TRUE NEGATIVE

D. accuracy of cancer detection by software, where abnormality was visible on radiograph		MDT-reported cancer REFERENCE STANDARD Positive	MDT-reported cancer REFERENCE STANDARD Negative
**Clinical report** TEST	**Positive**	51 TRUE POSITIVE	943 FALSE POSITIVE
	**Negative**	24 FALSE NEGATIVE	4687 TRUE NEGATIVE

E. accuracy of cancer detection by clinical reports, where abnormality was visible on radiograph		MDT-reported cancer REFERENCE STANDARD Positive	MDT-reported cancer REFERENCE STANDARD Negative
**Clinical report** TEST	**Positive**	60 TRUE POSITIVE	110 FALSE POSITIVE
	**Negative**	15 FALSE NEGATIVE	5520 TRUE NEGATIVE

For peer review only

Supplementary Table 1. Contingency tables. **A**: performance of the software compared to clinical reports. **B**: performance of the software compared to the MDT diagnosis of cancer. **C**: performance of the clinical reports compared to the MDT diagnosis of cancer. **D**: subgroup analysis; performance of the software compared to MDT diagnosis of cancer, where the abnormality was visible on the radiograph. **E**: subgroup analysis; performance of the clinical reports compared to MDT diagnosis of cancer, where the abnormality was visible on the radiograph.

For peer review only

Test	Suspicious nodule identified by software	Any suspicious abnormality on clinical report*
**Reference standard**	Cancer diagnosis by MDT decision, where the abnormality was visible on the radiograph	
**Sensitivity**	68.0% [56.2 - 78.3]	80.0% [69.2 - 88.4]
**Specificity**	83.3% [82.3 - 84.2]	98.0% [97.7 - 98.4]
**PPV**	5.1% [4.4 - 6.0]	35.3% [30.5 - 40.4]
**NPV**	99.5% [99.3 - 99.6]	99.7% [99.6 - 99.8]
**Accuracy**	83.0% [82.0 - 84.0]	97.8% [97.4 - 98.2]
**FPPI**	0.18	0.02

Supplementary Table 2. Subgroup analysis. Performance of the software and clinical reports against MDT diagnosis of cancer, where the abnormality was visible on the radiograph, with 95% confidence intervals. PPV = positive predictive value, NPV = negative predictive value, FPPI = false positives per image. *Includes nodules, masses and secondary features of malignancy.

For peer review only

943 false positive results	
**Normal anatomy (including variants and age-related changes)**	**659 cases (69.9%)**
Musculoskeletal (e.g. first rib, transverse processes of spine)	52.8%
Vessels (e.g. composite shadowing)	8.5%
Normal hila	29.9%
Nipple	7.0%
Foreign body (e.g. pacemaker)	3.8%
Other (e.g. bowel, age-related changes)	5.2%
Kyphosis or scoliosis	3.0%
**Technical factors**	**45 cases (4.8%)**
Projectional artefact (e.g. rotation)	73.3%
Expiratory or suboptimal inspiration	31.1%
**Non-cancerous pathology**	**294 cases (31.2%)**
Infective changes (e.g. consolidation, atelectasis)	40.8%
Benign-appearing nodule (e.g. hamartoma, granuloma)	13.3%
Fluid overload or pulmonary oedema	4.4%
Pulmonary fibrosis or scarring	15.6%
Pleural plaques	19.0%
Bronchiectasis	3.7%
Rib fracture	9.5%
Other	4.4%

Supplementary Table 3. Failure analysis of the 943 false positive results yielded by the ALND software. Broad causes of failure included normal anatomy, technical factors and non-cancerous pathology. For each of these, the relative proportion of specific underlying causes is provided. Note that in some cases multiple factors were identified.

For peer review only - http://bmjopen.bmj.com/site/about/guidelines.xhtml

Section / Topic	No.	Item	
TITLE / ABSTRACT			
	**1**	Identification as a study of AI methodology, specifying the category of technology used (e.g., deep learning)	**Yes**
	**2**	Structured summary of study design, methods, results, and conclusions	**Yes**
INTRODUCTION			
	**3**	Scientific and clinical background, including the intended use and clinical role of the AI approach	**Yes**
	**4**	Study objectives and hypotheses	**Yes**
METHODS			
Study Design	**5**	Prospective or retrospective study	**Yes**
	**6**	Study goal, such as model creation, exploratory study, feasibility study, non-inferiority trial	**Yes**
Data	**7**	Data sources	**Yes**
	**8**	Eligibility criteria: how, where, and when potentially eligible participants or studies were identified (e.g., symptoms, results from previous tests, inclusion in registry, patient-care setting, location, dates)	**Yes**
	**9**	Data pre-processing steps	**Yes**
	**10**	Selection of data subsets, if applicable	**N/A**
	**11**	Definitions of data elements, with references to Common Data Elements	**N/A**
	**12**	De-identification methods	**N/A**
	**13**	How missing data were handled	**N/A**
Ground Truth	**14**	Definition of ground truth reference standard, in sufficient detail to allow replication	**Yes**
	**15**	Rationale for choosing the reference standard (if alternatives exist)	**Yes**
	**16**	Source of ground-truth annotations; qualifications and preparation of annotators	**N/A**
	**17**	Annotation tools	**N/A**
	**18**	Measurement of inter- and intrarater variability; methods to mitigate variability and/or resolve discrepancies	**N/A**
Data Partitions	**19**	Intended sample size and how it was determined	**N/A**
	**20**	How data were assigned to partitions; specify proportions	**N/A**
	**21**	Level at which partitions are disjoint (e.g., image, study, patient, institution)	**N/A**
Model	**22**	Detailed description of model, including inputs, outputs, all intermediate layers and connections	**N/A**
	**23**	Software libraries, frameworks, and packages	**N/A**
	**24**	Initialization of model parameters (e.g., randomization, transfer learning)	**N/A**
Training	**25**	Details of training approach, including data augmentation, hyperparameters, number of models trained	**N/A**
	**26**	Method of selecting the final model	**N/A**
	**27**	Ensembling techniques, if applicable	**N/A**
Evaluation	**28**	Metrics of model performance	**N/A**
	**29**	Statistical measures of significance and uncertainty (e.g., confidence intervals)	**N/A**
	**30**	Robustness or sensitivity analysis	**N/A**
	**31**	Methods for explainability or interpretability (e.g., saliency maps), and how they were validated	**N/A**
	**32**	Validation or testing on external data	**N/A**
RESULTS			
Data	**33**	Flow of participants or cases, using a diagram to indicate inclusion and exclusion	**Yes**
	**34**	Demographic and clinical characteristics of cases in each partition	**Yes**
Model performance	**35**	Performance metrics for optimal model(s) on all data partitions	**Yes**
	**36**	Estimates of diagnostic accuracy and their precision (such as 95% confidence intervals)	**Yes**
	**37**	Failure analysis of incorrectly classified cases	**Yes**
DISCUSSION			

For peer review only

	**38**	Study limitations, including potential bias, statistical uncertainty, and generalizability	**Yes**
	**39**	Implications for practice, including the intended use and/or clinical role	**Yes**
OTHER INFORMATION			
	**40**	Registration number and name of registry	**N/A**
	**41**	Where the full study protocol can be accessed	**N/A**
	**42**	Sources of funding and other support; role of funders	**Yes**

Supplementary Table 4. CLAIM checklist

For peer review only
